# DRP1 contributes to head and neck cancer progression and induces glycolysis through modulated FOXM1/MMP12 axis

**DOI:** 10.1002/1878-0261.13212

**Published:** 2022-04-15

**Authors:** Tai‐Lin Huang, Chuang‐Rung Chang, Chih‐Yen Chien, Gong‐Kai Huang, Yi‐Fan Chen, Li‐Jen Su, Hsin‐Ting Tsai, Yu‐Sheng Lin, Fu‐Min Fang, Chang‐Han Chen

**Affiliations:** ^1^ Division of Hematology‐Oncology Department of Internal Medicine Kaohsiung Chang Gung Memorial Hospital and Chang Gung University College of Medicine Taiwan; ^2^ Institute of Biotechnology and Department of Medical Science National Tsing Hua University Hsinchu Taiwan; ^3^ Kaohsiung Chang Gung Head and Neck Oncology Group, Cancer Center Kaohsiung Chang Gung Memorial Hospital Taiwan; ^4^ Department of Otolaryngology Kaohsiung Chang Gung Memorial Hospital and Chang Gung University College of Medicine Taiwan; ^5^ Department of Anatomic Pathology Chang Gung Memorial Hospital Kaohsiung Taiwan; ^6^ Department of Orthopedic Surgery Chang Gung Memorial Hospital Kaohsiung Taiwan; ^7^ Department of Biomedical Sciences and Engineering, Education and Research Center for Technology Assisted Substance Abuse Prevention and Management, and Core Facilities for High Throughput Experimental Analysis National Central University Taoyuan County Taiwan; ^8^ Institute of Medicine Chung Shan Medical University Taichung Taiwan; ^9^ Department of Medical Research Chung Shan Medical University Hospital Taichung Taiwan; ^10^ State Key Laboratory of Optoelectronic Materials and Technologies School of Electronics and Information Technology Sun Yat‐Sen University Guangzhou China; ^11^ Department of Radiation Oncology Kaohsiung Chang Gung Memorial Hospital and Chang Gung University College of Medicine Taiwan

**Keywords:** DRP1, FOXM1, glycolysis, head and neck cancer, miR‐575, MMP12

## Abstract

Abnormal DRP1 expression has been identified in a variety of human cancers. However, the prognostic potential and mechanistic role of DRP1 in head and neck cancer (HNC) are currently poorly understood. Here, we demonstrated a significant upregulation of DRP1 in HNC tissues, and that DRP1 expression correlates with poor survival of HNC patients. Diminished DRP1 expression suppressed tumor growth and metastasis in both *in vitro* and *in vivo* models. DRP1 expression was positively correlated with FOXM1 and MMP12 expression in HNC patient samples, suggesting pathological relevance in the context of HNC development. Moreover, DRP1 depletion affected aerobic glycolysis through the downregulation of glycolytic genes, and overexpression of MMP12 in DRP1‐depleted cells could help restore glucose consumption and lactate production. Using ChIP‐qPCR, we showed that DRP1 modulates FOXM1 expression, which can enhance MMP12 transcription by binding to its promoter. We also showed that miR‐575 could target 3’UTR of *DRP1* mRNA and suppress DRP1 expression. Collectively, our study provides mechanistic insights into the role of DRP1 in HNC and highlights the potential of targeting the miR‐575/DRP1/FOXM1/MMP12 axis as a novel therapy for the prevention of HNC progression.

## Graphical abstract

This study reveals that high *DRP1* expression is correlated with poor survival in patients with head and neck cancer (HNC). Moreover, DRP1 modulates growth, metastasis and glycolysis of HNC cells via a FOXM1/MMP12 axis. We note that miR‐575 targets and degrades *DRP1* mRNA, inhibiting DRP1‐induced aggressive phenotypes. Our work highlights DRP1 as a potential therapeutic target for patients with HNC.

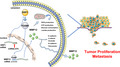

**Abbreviations**
DMEM: Dulbecco’s modified Eagle’s medium
EMPD: extramammary Paget’s disease
FOXM1: forkhead box protein M1
HNC: head and neck cancer
HRP: horseradish peroxidase
MEM: modified Eagle’s medium
MMP12: matrix metalloproteinase 12
MTT: 3‐(4,5‐dimethylthiazol‐2‐yl)‐2,5‐diphenyl‐tetrazolium bromide
PVD: polyvinylidene difluoride

## 1 Introduction

Head and neck cancer (HNC) was the seventh most common cancer worldwide in 2018 (890 000 new cases and 450 000 deaths) [1, 2] accounting for 3% of all cancers (51 540 new cases) and just over 1.5% of all cancer deaths (10 030 deaths) in the USA [3]. According to the Taiwan cancer registry annual report in 2017, HNC was the 6th most common cancer and the 5th most common cancer of cancer‐related death in Taiwan (7920 new cases and 2842 deaths). In addition, HNC in Taiwan was the 4th most common cancer and 4th most common cancer of cancer‐related death in males (7058 new cases and 2643 deaths). The incidence rate in men and women was 11 : 1 (Health Promotion Administration, Ministry of Health and Welfare, Taiwan; Cancer Registry Annual Report, 2017 Taiwan) [4]. Head and neck cancer was a highly aggressive solid tumor. The current curative treatment includes surgery, radiation therapy alone and radiation therapy combined with chemotherapy, depending on the stage of the lesion [5]. Despite tremendous strategy efforts, the prognosis of affected patients remains gloomy, particularly in those recurrent or metastatic HNC, with a definitely poor long‐term prognosis. Therefore, it is of great significance to explore tumor molecular markers which may effectively evaluate tumor screening, diagnosis, prognosis, recurrence and metastasis [6].

Mitochondria, dynamic organelles, play a crucial role in modulation of cellular function such as energy metabolism, calcium homeostasis, proliferation and differentiation to cell survival in cancer cells [7]. The mitochondria morphology changes constantly to maintain its shape, structure and function and this change is governed by fission and fusion in response to the microenvironment [8, 9]. Accumulating evidence demonstrates that cancer development is associated with mitochondria dynamics [8, 10, 11]. Four dynamin‐related GTPase proteins regulate the mitochondria dynamics: MFN1, MFN2, OPA1 and DRP1 [12]. Among these proteins, DRP1 is one of the crucial factors that related to tumor initiation and progression. Several studies have indicated that DRP1 is highly expressed in a variety of human cancers, such as lung, colorectal, pancreatic, breast cancers, cutaneous squamous cell carcinoma and glioblastoma, to promote tumorigenesis [13, 14, 15, 16, 17, 18]. In addition, DRP1 activation is also correlated with poor prognosis in glioblastoma. In extramammary Paget’s disease (EMPD), a positive correlation between DRP1 and clinical stages was revealed, indicating that DRP1 is a novel therapeutic target for EMPD [19]. Therefore, a major function for DRP1 in regulation of tumorigenesis has been suggested; however, there is a little information to demonstrate the pathological role of DRP1 in HNC. In addition, the molecular mechanisms of DRP1‐elicited tumor development are not entirely understood.

In the present study, we demonstrated the clinical significance of DRP1 in HNC. We also provided novel insight into a mechanism by which DRP1 mediated tumor growth and motility via FOXM1/MMP12 axis in HNC. These findings may represent a novel strategy for therapeutic interventions for HNC in the future.

## 2 Materials and methods

### 2.1 Patient samples

Written informed consent was obtained from all patients participating in this study. The study was approved by the Institutional Review Board of the Chang‐Gung Memorial Hospital (IRB Number: 201600645B0C501). We included pathology specimens from 92 patients in whom HNC had been diagnosed during the period from January 2012 to December 2012. All patients had undergone surgical resection, followed by observation or adjuvant treatment including irradiation alone or irradiation combined with chemotherapy, depending on the stage of the lesion. Formalin‐fixed, paraffin‐embedded tissue samples for all patients were obtained and analyzed retrospectively. Clinical and pathological factors were assessed by reviewing medical charts and pathology records. The patients with histologically confirmed HNC were included and the definitive histopathological diagnosis was performed by two certified pathologists based on the classification of the American Joint Committee on Cancer (AJCC), 7th edition. The adjuvant treatment post radical operation was radiotherapy alone or radiotherapy combined with cisplatin‐based chemotherapy if the patient was in the high risk recurrent or metastatic population. The date of diagnosis was defined as the date that the surgical resection of the malignancy was performed. The overall survival was defined as the period from the date of diagnosis to the date of the last follow‐up or the patient’s death. Disease‐free survival was defined as the period between the date of diagnosis and the date of tumor recurrence or metastasis. The study methodologies conformed to the standards set by the Declaration of Helsinki.

### 2.2 Cell lines and cultures

Human HNC cell lines SAS and HSC‐3 were obtained from the American Type Culture Collection (ATCC) and authenticated though STR typing. All experiments with cell lines were performed within 3 months after thawing. Logarithmically growing and mycoplasma‐negative cells were utilized for all experiments. SAS cells were cultured in Dulbecco’s modified Eagle’s medium (DMEM)/F12 (1 : 1) medium and HSC‐3 cells were cultured in modified Eagle’s medium (MEM) medium, supplemented with 10% FBS and 1% penicillin/streptomycin. All cells were cultured in an incubator at 37 °C and 5% CO_2_.

### 2.3 RNA extraction and quantitative RT‐PCR assay

Total RNA was prepared from indicated cells using a TRIZOL reagent. The cDNA was obtained using Reverse Transcription System reagent kit (Promega) according to the manufacturer’s protocols. QPCR assay was applied on an ABI 7500 system using a SYBR Premix Ex Taq kit (TaKaRa). A 2−ΔΔCT method was used to calculate the relative expression of each gene. The GAPDH was used as an internal control. The primer sequences were as follows: *DRP1* (forward: 5’‐ AAGAACCAACCACAGGCAAC‐3’, reverse: 5’‐GTTCACGGCATGACCTTTTT‐3’), *FOXM1* (forward: 5′‐CATTAAGGAAACGCTGCCCA‐3’, reverse: 5′‐GGT TCTGAACTGAGGAGCCT‐3′), *MMP12* (forward: 5′‐GCTGTCACTACCGTGGGAAA‐3′, reverse: 5′‐GGCAAGGTTGGCCATAAGGA‐3′), and *GAPDH* (forward: 5’‐CACCAACTGGGACGACATG‐3’, reverse: 5’‐ GCACAGCCTGGATAGCAAC‐3’).

### 2.4 Cell transfection

The negative control, *siDRP1*, *siFOXM1*, mimics negative control, miR‐575 mimics, negative control inhibitor, and miR‐575 inhibitor were synthesized and purified by Dharmacom. HNC cells were seeded in six‐well plates and grown overnight. Next day, siRNA or miRNA were transfected to cells using LipofectamineTM 3000 reagent following the manufacturer’s instructions. After 24 h, the expressions of target genes were determined by QPCR or western blotting.

### 2.5 Western blot assay

Cells were lysed using RIPA buffer (50 mm Tris‐HCl (pH = 7.4), 150 mm NaCl, 1% NP‐40) and quantified by Bradford reagent. Proteins were loaded into 10% SDS‐PAGE, and then transferred to polyvinylidene difluoride (PVDF) membranes (Millipore, Burlington, MA, USA). The membranes were blocked with TBST containing 5% skimmed milk for 1 h at room temperature, and then incubated with the corresponding primary antibodies anti‐DRP1 (1 : 1000, Cell Signaling #8570), anti‐FOXM1 (1 : 1000, GeneTex GTX100276), anti‐MMP12 (1 : 1000, Novus NBP1‐31225) and β‐actin (1 : 5000, 8H10D10, Cell Signaling Technology, Danvers, MA, USA) at 4 °C overnight. Next day, the membranes were washed with TBST three times and incubated with secondary antibody at room temperature for 1 h. The protein signals were detected by ECL Detection Reagent. Quantification of the band intensity was performed using imagej software.

### 2.6 Dual‐luciferase reporter assay

The 3’UTR of wild‐type or mutant *DRP1*, an assumed *miR‐575* binding site, was ligated into the pmirGLO luciferase vector (Promega, Madison, WI, USA). HNC cells were co‐transfected with *miR‐575* mimic (or negative control) and the *DRP1*‐wild‐type or *DRP1*‐mutated by Lipofectamine 3000 (Life Technologies, Carlsbad, CA, USA). After transfection 36 h, the luciferase activities were detected using a dual‐luciferase reporter system (Promega) according to the manufacturer’s instruction. A 600‐bp DNA fragment spanning +1 to −600 of MMP12 promoter containing FOXM1 binding site was amplified and cloned into pGL3 basic vector. The promoter sequence was validated by sequencing. For examining promoter activity, cells were cotransfected with pGL3‐basic vector or pGL3‐*MMP12* and internal control pRL‐TK plasmid (250 ng·mL^−1^) with/without Flag‐FOXM1 expression vector using Lipofectamine 3000. Following cell lysis (36 h post‐transfection), Firefly and Renilla luciferase activities were evaluated in the Firefly/Renilla Dual Luciferase Reporter Assay System according to the manufacturer’s instructions (Promega). All reporter gene assays were performed in triplicate and repeated at least 3 times.

### 2.7 Cell growth assay

CCK8 (Cell counting kit‐8) assay was performed to detect relative cell viability according to the manufacturer’s protocol. Briefly, cultured cells were incubated with 10 μL·well^−1^ of CCK‐8 solution at 37 °C for 2 h. The optical density (*A*) value at 450 nm was then measured with a microplate reader.

### 2.8 Migration and invasion assays

Cell migration and invasion assays were performed using Transwell chambers (BD Biosciences, Franklin Lakes, NJ, USA). For migration assay, 5 × 10^3^ cells were diluted in 200 μL culture medium containing 10% FBS and then seeded in upper chamber of a Transwell. The medium volume of bottom chamber was 600 μL. For invasion assay, The Transwell was coated with Matrigel (100 μg·mL^−1^) after precooling on ice and incubating the plate in 37 °C for 2 h followed by aspirating the unsolidified Matrigel. Next, 1 × 10^4^ cells were diluted in 200 μL culture medium containing 10% FBS and then seeded in the upper chamber of a Transwell. The medium volume of bottom chamber was 600 μL. After incubation at 37 °C for 24 h, the migratory or invasive cells were fixed in methanol and stained with 0.1% crystal violet. For quantification, the numbers of migrated or invasive cells were calculated by counting at least five random separate fields for determination of the ratio of the experimental samples to the control samples.

### 2.9 Wound healing assay

Cells were plated into six‐well plates and incubated at 37 °C until confluence reached about 90%. Next, we scratched the monolayer with a 10‐µL pipette tip to create a mechanical wound. After 24 h, the percentage of the wound closure area relative to the original area was evaluated by microscopy and imagej software.

### 2.10 Immunohistochemical staining

Paraffin‐embedded HNC specimens were cut at 4 μm, and rehydration was performed with xylene. The slides were blocked with 3% H_2_O_2_ and then with 5% BSA for 2 h at room temperature. After incubation with anti‐DRP1 overnight at 4 °C, the slides were incubated with horseradish peroxidase (HRP)‐conjugated secondary antibodies for 1 h at room temperature, developed with a DAB staining kit and counterstained with hematoxylin. The scores were independently rendered by two pathologists. The expression levels were scored were determined as proportion of immunopositive staining area (0%, 0; < 10%, 1; 10–50%, 2; 50–75%, 3; 75–100%, 4) multiplied by intensity staining (0, negative; 1, weak; 2, moderate; 3, intense) [20, 21]. Specimens scoring beyond 3 were considered positive expressions [22].

### 2.11 ChIP assay

The DNA/protein complex was extracted using EZ‐CHIP KIT (Millipore). The samples were incubated with anti‐FOXM1 antibody or anti‐IgG antibody. Protein and DNA were de‐crosslinked with 5 m NaCl and proteinase K. DNA was purified and quantified by qPCR.

### 2.12 ELISA assay

The conditioned medium was collected from SAS and HSC‐3 cells transfected with negative control or *siDRP1* for 24 h. Extracellular MMP‐12 protein level was determined using a chemiluminescent microparticle immunoassay according to the manufacturer’s instructions.

### 2.13 PCR array

Human Tumor Metastasis PCR array was obtained from Life Technologies Corporation and performed according to the manufacturer’s instructions. The amplification reaction and the results were analyzed using the rq manager software.

### 2.14 Glucose consumption and lactate production

The culture medium of HNC transfectants was collected for the determination of glucose consumption and lactate production. Glucose and lactate levels were measured using a Glucose Uptake‐Glo^TM^ Assay Kit (Promega) and a lactate‐Glo^TM^ Assay Kit (Promega), respectively, according to manufacturer’s protocol.

### 2.15 Cellular ROS analysis

The intracellular ROS levels of each group were measured by the ROS detection assay Kit (BioVision, Milpitas, CA, USA) following the manufacturer’s instructions. Cells were stained with fluorescence dye DCFH‐DA (10 μm) for 30 min in a darkroom. The fluorescent signal was measured using Ex/Em 495/529 nm in a microplate reader.

### 2.16 ATP assay

The cellular ATP content was detected using the ATP Bioluminescence Assay Kit (BioVision) according to manufacturer’s instructions. A 5‐µL sample was added to 95 µL of the ATP reaction mixture and readings were taken at 570 nm.

### 2.17 Animal study

Male BALB/c‐nu mice were obtained from Bio‐LASCO Taiwan Co., Ltd and housed under specific pathogen‐free conditions with 12 h light/12 h dark cycle at 24 ± 2 °C temperature, and a relative humidity of 50 ± 10% according to the guidelines of the Animal Care Committee at the Kaohsiung Chang‐Gung Memorial Hospital, Taiwan. Male BALB/c‐nu mice were subcutaneously injected with 1 × 10^6^ SAS cells to establish HNC tumors. When tumors reached a volume of 100 mm^3^, mice were randomly divided into two groups and given Mdivi‐1 (15 mg·kg^−1^) [23] by intraperitoneal injection for 20 days. Tumor size was measured once every 5 days. Five weeks later, the mice were sacrificed, and tumor size was measured again. For the tumor‐bearing DRP1 expression xenograft model, SAS cells stably transfected with *shDRP1* or shcontrol (1 × 10^6^) were subcutaneously injected into the nude mice. Tumor volume was monitored every 5 days. Four weeks later, the mice were sacrificed. Volumes were calculated using the following formula: Volume (mm^3^) = [width^2^ (mm^2^) × length (mm)]/2. With respect to *in vivo* tumor metastasis experiment, 5‐week‐old BALB/c‐nu mice were injected with 5 × 10^5^ cells via the tail vein. Starting the next day, mice were treated daily with 15 mg·kg^−1^ Mdivi‐1 by intraperitoneal injection. For DRP1 inhibition of tumor metastasis *in vivo*, DRP1‐depleted SAS or shcontrol‐SAS (5 × 10^5^) cells were injected into the tail vein of nude mice. Six weeks later, lungs were excised and kept in formalin. The number of metastatic nodules in lung was observed under microscope via H&E staining. The animal study was approved by the Committee on the Chang‐Gung Memorial Hospital.

### 2.18 Statistical analysis

GraphPad prism 8 was used to plot and for biostatistical analysis in the study. Correlation between patient survival and gene expression was evaluated by Kaplan–Meier analysis and log‐rank test. Spearman’s test was used for correlation analysis. Student’s *t*‐test, Mann–Whitney nonparametric test or one‐way ANOVA was performed for comparison of means between the two groups. All data are presented as means ± SD with three independent experiments.

## 3 Results

### 3.1 DRP1 expression is correlated with poor outcome in HNC

**Fig. 1:**
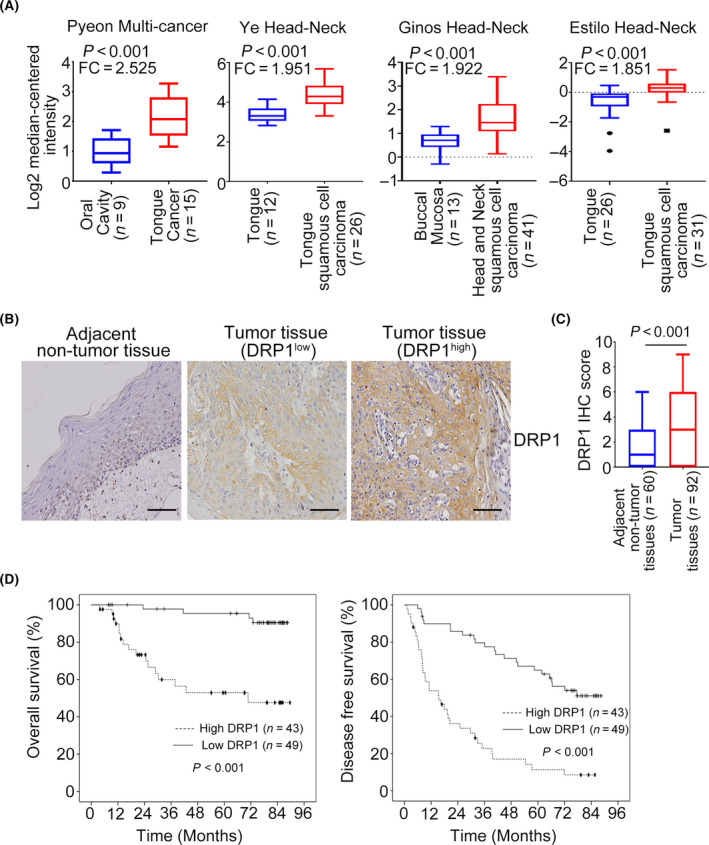
DRP1 expression is upregulated in HNC and is associated with poor outcomes. (A) The *DRP1* mRNA expression in HNC samples from Oncomine datasets. Data are presented as mean  ±  SD. Significance is calculated using *t*‐test. (B) Representative images of immunohistochemical staining for DRP1 in adjacent nontumor tissues and tumor tissues of HNC. Scale bar: 100 µm. (C) Average staining scores for DRP1 expression in HNC tumor tissues and adjacent nontumor tissues. Data are presented as mean  ±  SD. Significance is calculated using unpaired *t*‐test. (D) DRP1 expression in overall and disease‐free survival was assessed in patients with HNC using Kaplan–Meier analyses. *P*‐values were determined using the log‐rank test.

To explore the role of *DRP1* in HNC, we first analyzed the expression of DRP1 in public databases from Oncomine databases. Results showed that the mRNA level of *DRP1* was significantly higher in primary HNC tumor tissues than in normal tissues (Figs 1A and S1A), suggesting that the high *DRP1* expression is associated with development of HNC.

To investigate further the clinically significance of DRP1 in HNC, immunohistochemical staining was performed. Descriptive clinicopathological characteristics of the 92 patients in the study are illustrated in Table 1. The representative immunohistochemical staining images of DRP1 are shown and indicated that DRP1 was significantly higher in tumor tissues of HNC than in adjacent nontumor tissues (Fig. 1B). Statistical analysis demonstrated a relatively higher IHC score of DRP1 in tumor tissues than in paired adjacent nontumor tissues (Fig. 1C). Next, we investigated the association between DRP1 expression and clinical characteristics. High DRP1 expression was only associated with lymph node stage and clinical stage (Table 2). Survival analysis indicated that DRP1 overexpression was dramatically correlated with worse overall survival and disease‐free survival (Fig. 1D). In addition, the univariate and multivariate Cox regression analysis revealed that DRP1 overexpression was an independent factor for overall survival and disease‐free survival (Table 3). Collectively, these data demonstrated that increased DRP1 expression is a prognostic factor for HNC.

**Table 1:** Characteristics of 92 surgery patients with HNSCC.

Variable	*n*	%
Age		
Median		55
Mean		54.7
≤60	64	69.6
>60	28	30.4
Gender		
Male	86	93.5
Female	6	6.5
Cancer site		
Oral	65	70.7
Oropharynx	18	19.6
Hypopharynx	6	6.5
Larynx	3	3.3
pT status		
T1	22	24.2
T2	33	36.2
T3	7	7.7
T4	29	31.9
pN status		
N0	51	55.4
N1	18	19.6
N2	22	23.9
N3	1	1.1
Clinical stage 7th AJCC stage		
I	14	15.2
II	19	20.7
III	19	20.7
IV	40	43.4
Adjuvant Tx		
No	40	43.5
Yes	52	56.5
DRP1 expression		
Low	49	53.3
High	43	46.7

**Table 2:** Association between DRP1 expression and clinicopathological parameters in 92 surgery patients with HNSCC.

Parameters		DRP1 expression			*P*‐value
		All case	Low	High	
		*n* (%)	*n* (%)	*n* (%)	
Age, years	≤60	64 (69.6)	32 (65.3)	32 (74.4)	0.343
	>60	28 (30.4)	17 (34.7)	11 (25.6)	
Gender	Male	86 (93.5)	47 (95.9)	39 (90.7)	0.413^a^
	Female	6 (6.5)	2 (4.1)	4 (9.3)	
Cancer site	Oral	65 (70.6)	34 (69.4)	31 (72.1)	0.671^a^
	Oropharynx	18 (19.6)	11 (22.4)	7 (16.3)	
	Hypopharynx and larynx	9 (9.8)	4 (8.2)	5 (11.6)	
pT status	T1–T2	55 (60.4)	33 (67.3)	22 (52.4)	0.146
	T3–T4	36 (39.6)	16 (32.7)	20 (47.6)	
pN status	N0	51 (55.4)	32 (65.3)	19 (44.2)	0.042^*^
	N1–N3	41 (44.6)	17 (34.7)	24 (55.8)	
Clinical stage	I–II	33 (35.9)	22 (44.9)	11 (25.6)	0.05^*^
	III–IV	59 (64.1)	27 (55.1)	32 (74.4)	
Adjuvant Tx	No	40 (43.5)	25 (51.0)	15 (34.9)	0.119
	Yes	52 (56.5)	24 (49.0)	28 (65.1)	

^a^ *P*‐value for Fisher’s exact test. *Significant: *P* ≤ 0.05.

**Table 3:** Univariate and multivariate analysis of DRP1 expression and overall survival, and disease‐free survival.

Variable	Overall survival				Disease‐free survival			
	Univariate analysis		Multivariate analysis		Univariate analysis		Multivariate analysis	
	HR (95% CI)	*P*‐value	aHR* (95% CI)	*P*‐value	HR (95% CI)	*P*‐value	aHR* (95% CI)	*P*‐value
Age (≤ 60 vs > 60)	3.1 (0.9–10.5)							
Gender (female vs male)	2.7 (0.6–11.7)	0.182	4.3 (0.92–20.4)	0.064	1.6 (0.6–4.3)	0.39		
Cancer site								
Oropharynx vs oral	0.7 (0.2–2.4)	0.589			1.2 (0.6–2.3)	0.571	1.6 (0.8–3.2)	0.149
Hypopharynx & larynx vs oral	1.0 (0.2–4.2)	0.956			1.7 (0.8–3.7)	0.157	1.5 (0.7–3.3)	0.303
pT status (T3–T4 vs T1–T2)	2.2 (0.9–5.2)	0.071	2.0 (0.8–4.7)	0.129	1.8 (1.04–3.0)	0.033	2.0 (1.2–3.5)	0.013
pN status (N1–N3 vs N0)	0.7 (0.3–1.8)	0.48			1.0 (0.6–1.7)	0.883		
Clinical stage (III–IV vs I–II)	1.3 (0.5–3.3)	0.559			1.1 (0.6–1.8)	0.81		
Adjuvant Tx (Yes vs No)	1.1 (0.5–2.6)	0.869			0.8 (0.5–1.3)	0.312		
DRP1 expression (High vs low)	5.8 (2.1–16.0)	0.001	6.5 (2.2–18.5)	0.001	3.3 (1.9–5.6)	< 0.001	3.5 (2.0–6.1)	< 0.001

* aHR, HR adjusted variables where *P*‐value of univariate analysis was < 0.200.

### 3.2 DRP1 inhibition reduces cell growth in HNC

**Fig. 2:**
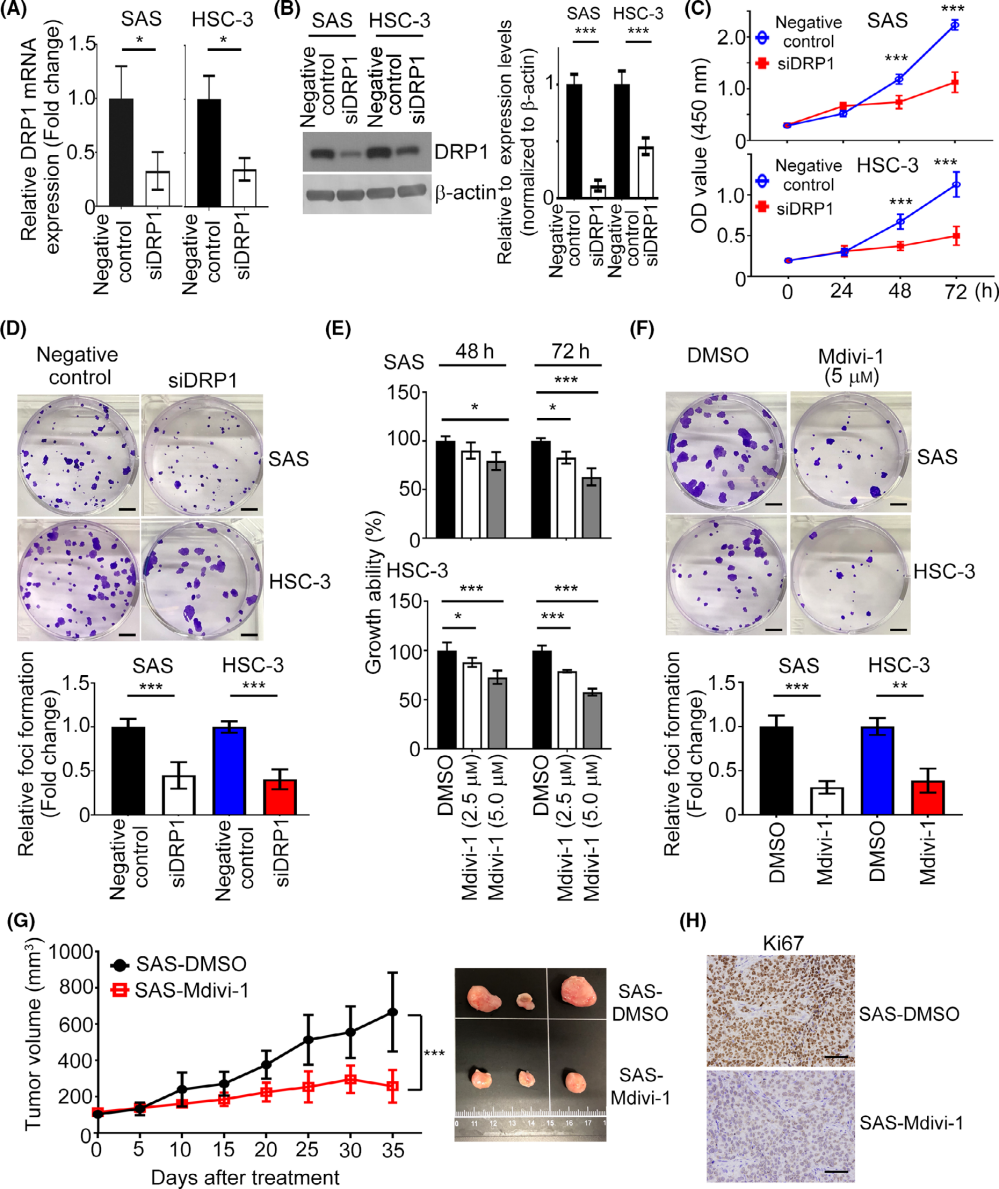
Inhibition of DRP1 retards cell growth in HNC. (A,B) The mRNA and protein expression levels of DRP1 in both SAS and HSC‐3 cells were determined by QPCR and western blotting. Quantification of relative DRP1 expression is shown. (C, D) The effect of DRP1 on cell proliferation was examined by CCK8 and foci formation assays. Scale bar: 500 µm. (E, F) The impact of Mdivi‐1 on cell growth was determined by CCK8 and foci formation assays. Scale bar: 500 µm. (G) Cells stimulated with Mdivi‐1 and control groups were injected into the right flank of nude mice for 4 weeks; *n* = 5 per group. The tumor volumes were measured. (H) KI67 staining indicated cell growth in tumor cells. Scale bar: 50 µm. All data presented as mean  ±  SD of three independent experiments. Significance calculated in (A,B,D,F,G) using *t*‐test. Significance calculated in (C) and (E) using one‐way ANOVA followed by Tukey’s multiple comparison’s test. * *P* < 0.05, ** *P* < 0.01, *** *P* < 0.001.

Next, we determined the biological functions of DRP1 in HNC cells. SAS and HSC‐3 cells were transfected with *DRP1*‐mediated siRNA and the mRNA, and protein expressions of DRP1 were examined by QPCR and western blotting (Fig. 2A,B). The 3‐(4,5‐dimethylthiazol‐2‐yl)‐2,5‐diphenyl‐tetrazolium bromide (MTT) and colony formation assays indicated that DRP1 inhibition dramatically decreased the ability of cell proliferation (Fig. 2C) and frequency of foci formation (Fig. 2D) in HNC cells. Moreover, treatment with SAS and HSC‐3 cells with Mdivi‐1, a small‐molecule inhibitor targeting *DRP1*, not only significantly reduced the DRP1 protein expression but also cell growth and colony numbers (Figs [Link], [Link], [Link], [Link], [Link], [Link], [Link]B, Fig. 2E,F). In an *in vivo* study, tumors derived from SAS cells treated with Mdivi‐1 were much smaller and lighter than those in the control group. Tumor formation was observed only in 3/5 of mice injected with SAS‐treated with Mdivi‐1 but was observed in 4/5 of mice in the control group (Fig. 2G). KI67 staining was performed to evaluate the proliferation of tumor cells (Fig. 2H). We next assessed the *in vivo* role of DRP1. The results showed that xenograft stably depletion of DRP1 grew slower and dramatically decreased in tumor volume, compared with control groups (Fig. [Link], [Link], [Link], [Link], [Link]C). The IHC assay demonstrated that the expression of DRP1 was downregulated in the shDRP1‐depleted tumor tissue compared with the shcontrol group (Fig. [Link], [Link], [Link], [Link]D). These data could imply that DRP1 expression is involved in cell proliferation in HNC cells.

### 3.3 DRP1 suppression prevents cell migration and invasion in HNC

**Fig. 3:**
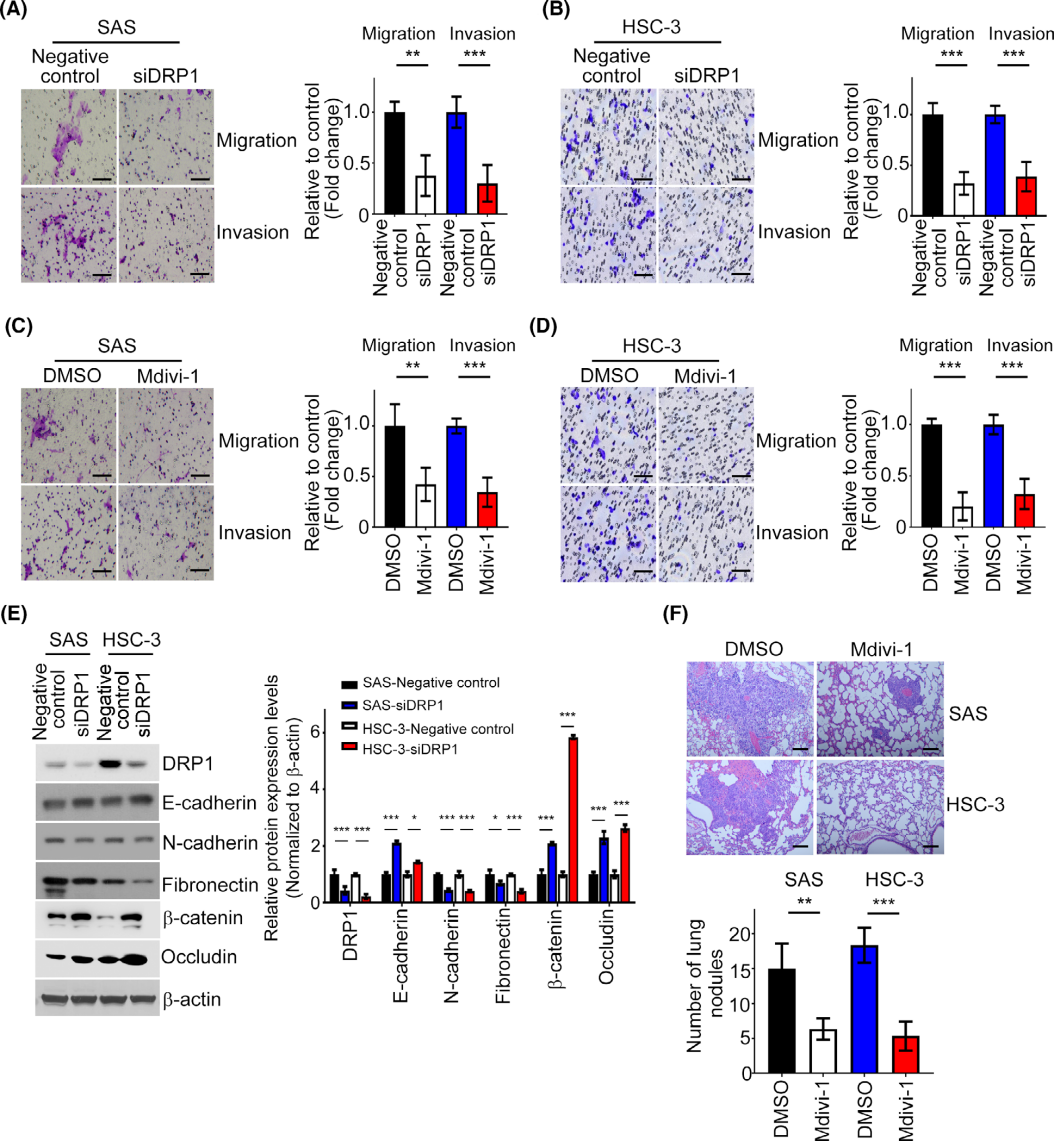
DRP1 depletion restrains cell metastasis in HNC. (A, B) The Transwell assay was performed to examine the effect of DRP1 on cell migration and invasion in SAS and HSC‐3 cells. The representative images and the fold changes of cell migration and invasion are presented. Scale bar: 100 µm. (C, D) Migration and invasion assays of SAS and HSC‐3 cells treated with Mdivi‐1. The representative images and the fold changes of cell migration and invasion are presented. Scale bar: 100 µm. (E) DRP1, E‐cadherin, N‐cadherin, fibronectin, β‐catenin and Occludin protein expressions were determined in SAS/negative control, SAS/ *siDRP1*, HSC‐3/negative control, and HSC‐3/ *siDRP1* by western blotting. (F) HE staining was performed to demonstrate the tumor nodule in the lungs; *n* = 4 per group. Representative images and statistical analyses are shown. Scale bar: 50 µm. All data are presented as mean  ±  SD of three independent experiments. Significance was calculated using *t*‐test. ** *P* < 0.01, *** *P* < 0.001.

As clinicopathological results revealed that DRP1 expression was associated with metastatic characteristics in HNC, the impact of DRP1 on HNC cell motility was investigated. Transwell assays showed that the migratory and invasive abilities of SAS and HSC‐3 cells were decreased, and endogenous DRP1 was depleted, compared with the negative control group (Fig. 3A,B). Similarly, the numbers of migrated and invasive cells also decreased in HNC cells treated with Mdivi‐1 (Fig. 3C,D). Wound‐healing assay also indicated that DRP1 inhibition reduced the migration abilities of SAS and HSC‐3 cells and interfered the closure of wound width of the cell (Fig. [Link], [Link], [Link], [Link], [Link], [Link], [Link]A,B). This is consistent with the results above that SAS or HSC‐3 cells treated with Mdivi‐1 impeded wound closure (Fig. [Link], [Link], [Link], [Link], [Link]C,D). Moreover, the protein expression levels of E‐cadherin, β‐catenin and Occludin were upregulated and N‐cadherin and fibronectin downregulated in DRP1‐depeted SAS and HSC‐3 cells in western blotting analysis (Fig. 3E). An *in vivo* tumor metastasis model illustrated that fewer lung nodules were observed in mice injected with SAS or HSC‐3 cells stimulated with Mdivi‐1 (Fig. 3F). Additionally, the DRP1‐depleted/SAS group had fewer lung metastasis compared with the shcontrol group (Fig. [Link], [Link], [Link], [Link]E,F), indicating that DRP1 knockdown prevented the distant metastasis of HNC cells. Collectively, these data suggest that DRP1 elicits cell motility via EMT process in HNC cells.

### 3.4 *DRP1* is targeted by miR‐575 in HNC

**Fig. 4:**
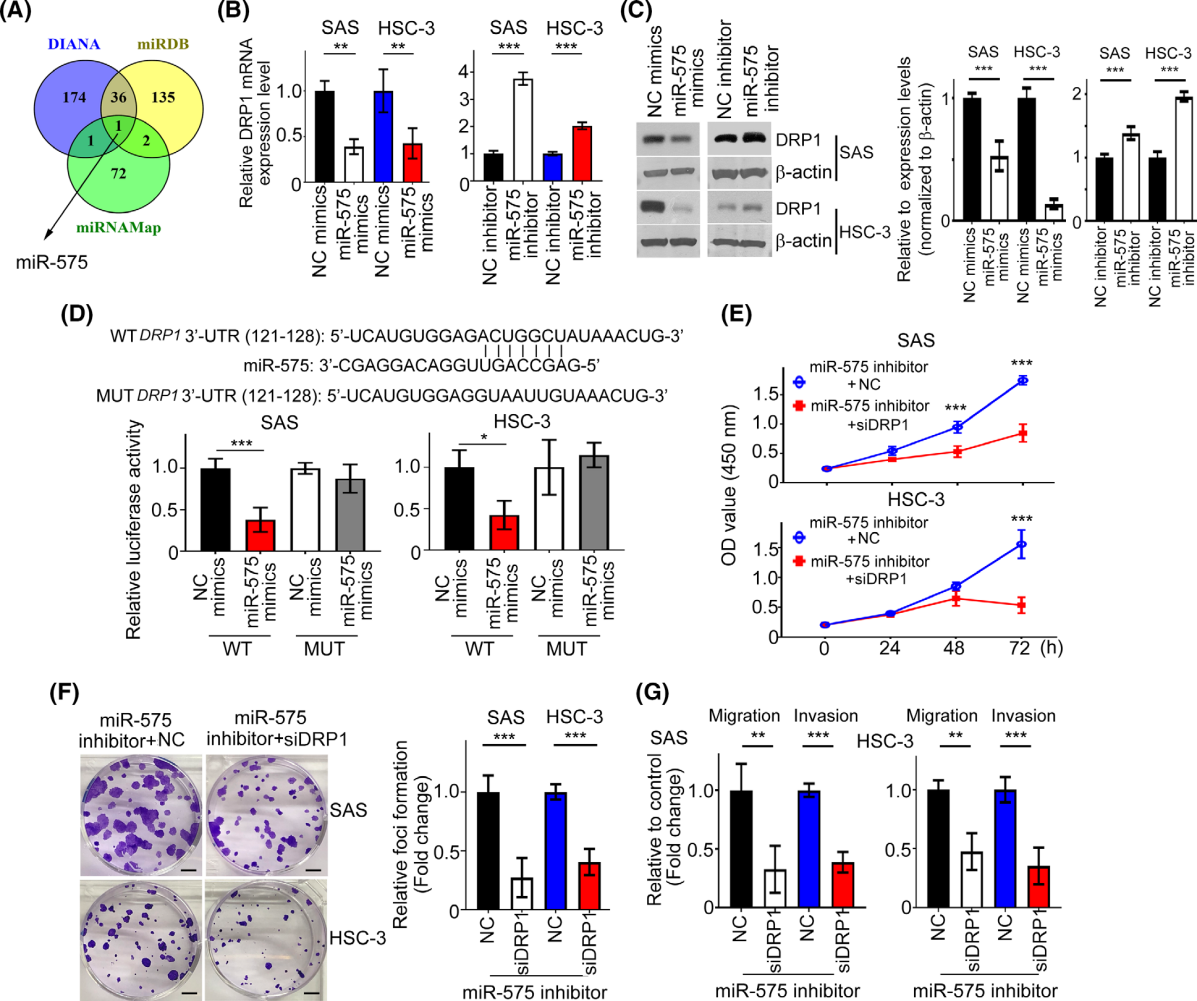
DRP1 targeted by miR‐575. (A) A drawn Venn diagram was used to identify miRNA among three cohort profile datasets. (B) The mRNA expression profiles of *DRP1* were determined by QPCR in SAS and HSC‐3 cells transfected with miR‐575 mimics or inhibitors. (C) SAS and HSC‐3 cells were transfected with miR‐575 mimics or inhibitor for 36 h. DRP1 protein was investigated by western blotting. Quantification of relative DRP1 expression was shown. (D) Luciferase reporter assays were performed to demonstrate the influence of miR‐575 on the activity of *DRP1* mRNA 3’UTR. (E– G) Cells expressing miR‐575 inhibitor were transfected with *siDRP1* or NC for 24 h. Cell proliferation, migration and invasion were examined by MTT, colony formation and Transwell assays. NC, negative control. Scale bar: 500 µm. All data are presented as mean  ±  SD of three independent experiments. Significance was calculated using *t*‐test. In (E), statistical analyses were performed using one‐way ANOVA followed by Tukey’s multiple comparison’s test. * *P* < 0.05, ** *P* < 0.01, *** *P* < 0.001.

To elucidate how DRP1 modulates cell proliferation and motility in HNC cells, we performed Venn diagram analysis of predicted *DRP1* targets from three independent databases: miRDB, DIANA and miRNAMap. At the intersection of these databases, we found that miR‐575 was predicted to be a potential target of *DRP1* (Fig. 4A). Next, miR‐575 was introduced to SAS and HSC‐3 cells and we assessed the mRNA expression level of *DRP1*. Our data indicated that miR‐575 significantly decreased *DRP1* mRNA expression level in HNC cells (Fig. 4B). In contrast, suppression of miR‐575 by its specific inhibitor resulted in a dramatic increase of *DRP1* mRNA (Fig. 4B). Accordantly, the protein expression level of DRP1 was downregulated by miR‐575 mimics but upregulated by miR‐575 inhibitor in SAS and HSC‐3 cell lines (Fig. 4C). Furthermore, enforced miR‐575 mimics remarkably inhibited the activity of wild‐type *DRP1* 3’UTR but not the mutant 3’UTR by dual luciferase assays, and a putative site for the binding of miR‐575 in *DRP1* 3’UTR was shown (Fig. 4D). Next, the effect of DRP1 on miR‐575‐raised aggressive phenotypes was assessed. MTT, colony formation, migration and invasion assays showed that suppression of DRP1 decreased growth, foci formation and motility in SAS and HSC‐3 cells with expression of miR‐575 inhibitor (Fig. 4E‐G). Decreased cell growth, colony numbers and motility of HNC cells were also observed in miR‐575 mimics transfectants with DRP1 knockdown (Fig. [Link], [Link], [Link], [Link], [Link], [Link], [Link]A–C). Collectively, these data demonstrated that DRP1 contributes to the tumor‐suppressive activity of miR‐575 in HNC cells.

### 3.5 Identification of downstream targets of DRP1 in HNC

**Fig. 5:**
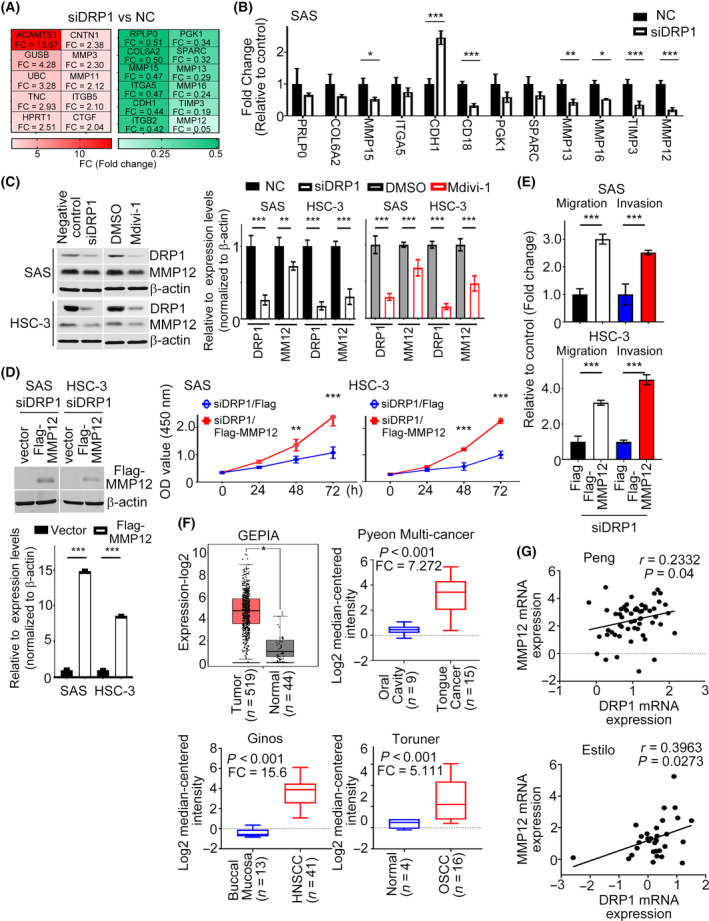
MMP12 is one of the targets of DRP1 in HNC cells. (A) Heat‐map showing relative alteration of target genes belonging to EMT molecules using QPCR array analysis of SAS cell transfected with *siDRP1* compared with the negative control. Red: upregulation; green: downregulation. (B) QPCR was analyzed to validate the expressions of target genes from (A). (C) Impact of DRP1 knockdown or Mdivi‐1 on MMP12 protein expression were demonstrated in SAS and HSC‐3 cells. Quantification of relative DRP1 and MMP12 expressions are shown. (D,E) Cell growth and motility were evaluated in DRP1‐depleted cells transfected with MMP12 using MTT and Transwell assays. Quantification of relative Flag‐MMP12 expression is shown. (F) Studies from GEPIA and Oncomine datasets present the increase of *MMP12* mRNA in HNC samples. (G) A positive correlation between *DRP1* mRNA and *MMP12* mRNA was found in the Oncomine cohort (Peng database, *n* = 41; Estilo database, *n* = 31). All data are presented as mean  ±  SD of three independent experiments. Significance was calculated using *t*‐test. In (D) (cell growth) and (F) (GEPIA), statistical analyses were performed using one‐way ANOVA followed by Tukey’s multiple comparison’s test and Wilcoxon signed‐rank test, respectively. * *P* < 0.05, ** *P* < 0.01, *** *P* < 0.001.

To determine the underlying mechanism towards HNC development via DRP1, a pathway‐focused gene expression pattern was evaluated using *DRP1*‐depleted SAS cells. Of the different genes represented on PCR arrays related to extracellular matrix, using a fold change of > 2 or < 0.5 as the cutoff criteria, suppression of *DRP1* expression resulted in downregulation of *RPLP0*, *COL6A2*, *MMP15*, *ITGA5*, *CDH1*, *ITGB2*, *PGK1*, *SPARC*, *MMP13*, *MMP16*, *TIMP3* and *MMP12*, and upregulation of *ADAMTS1*, *GUSB*, *UBC*, *TNC*, *CNTN1*, *HPRT1*, *MMP3*, *MMP11*, *ITGB5* and *CTGF* genes, compared with the negative control (Fig. 5A). Here, we focused on the downregulation groups. Upon verification using SAS and HSC‐3 cells transfected with *DRP1* siRNA, endogenous *MMP12* mRNA expression remained consistently and significantly decreased (Figs 5B and [Link], [Link], [Link], [Link], [Link], [Link], [Link]A). In addition, we performed a luciferase reporter assay and found that luciferase activity was lower in HNC cells coexpressing *siDRP1* and *MMP12* promoter, suggesting that DRP1 positively regulates the promoter activity of *MMP12* (Fig. [Link], [Link], [Link], [Link], [Link]B). The impact of MMP12 protein expression in DRP1‐depleted HNC cells or HNC cells treated with Mdivi‐1was also validated by western blotting and ELISA (Figs 5C and [Link], [Link], [Link], [Link]C). Functionally, overexpressing MMP12 in DRP1‐depleted SAS and HSC‐3 cells reversed *siDRP1*‐inhibited cell growth, migration, and invasion, compared with siDRP1 cells (Fig. 5D,E). According to the expression of *MMP12* mRNA in GEPIA and Oncomine databases, *MMP12* was more highly expressed in tumor samples of HNC patients than in normal tissues (Figs 5F and [Link], [Link], [Link], [Link], [Link], [Link], [Link]A). Importantly, *DRP1* mRNA expression was positively correlated with the expression of *MMP12* in both GEPIA and Oncomine HNC samples (Figs 5G and [Link], [Link], [Link], [Link], [Link]B). Thus, DRP1‐affected cell proliferation and motility is required for MMP12 expression in HNC.

### 3.6 DRP1 potentiates glycolysis via MMP12 in HNC

**Fig. 6:**
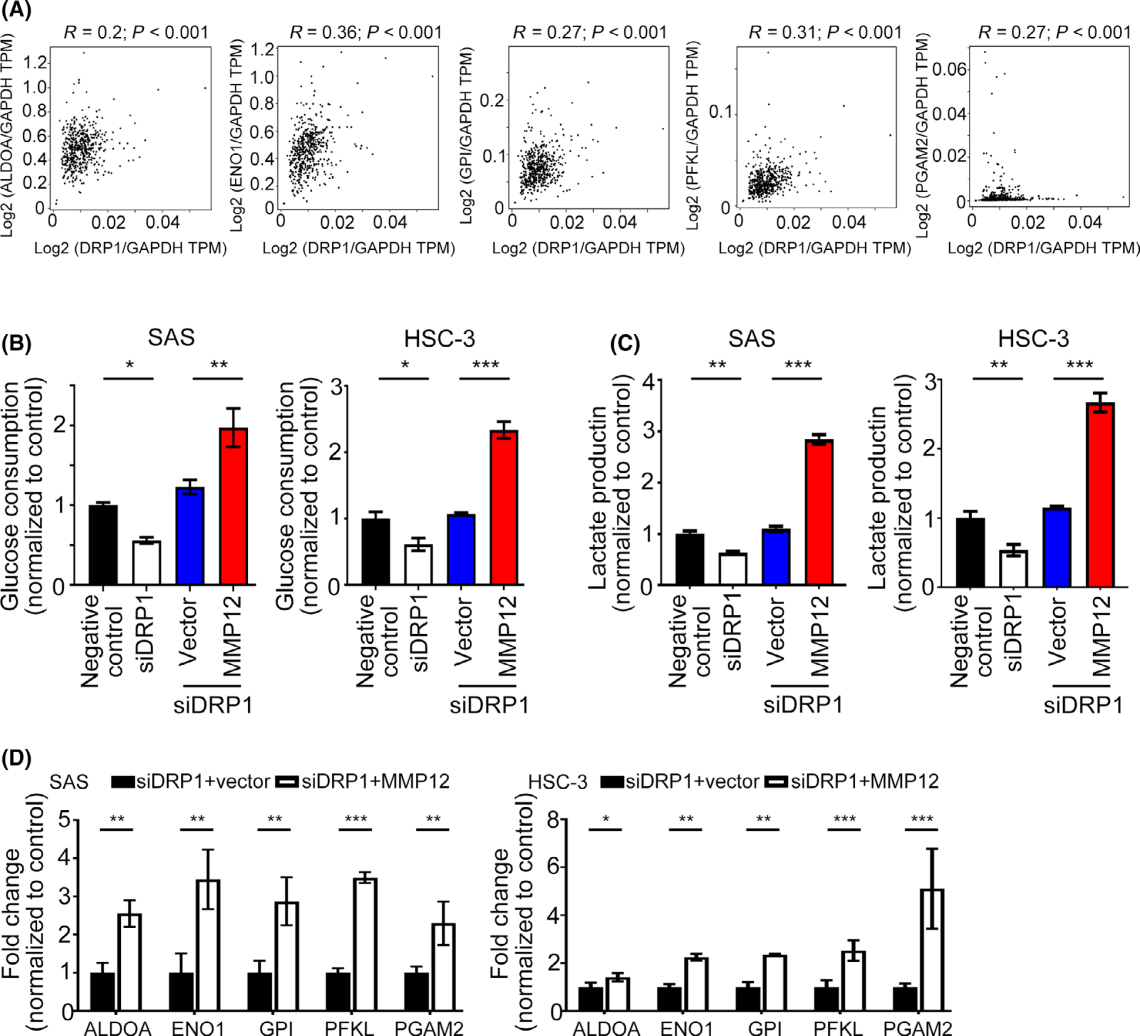
DRP1 potentiates glycolysis in HNC cell that is required for MMP12. (A) The correlation between *DRP1* and glycolytic molecules was assessed from the GEPIA database (*n* = 519). (B,C) Glucose consumption and lactate production were measured in *siDRP1*‐SAS and ‐HSC‐3 cells transfected with MMP12 or vector alone. (D) The transcriptional levels of glycolytic molecules were examined by QPCR in *siDRP1*‐SAS and ‐HSC‐3 cells transfected with MMP12 or vector alone. All data are presented as mean  ±  SD of three independent experiments. Significance calculated using *t*‐test. * *P* < 0.05, ** *P* < 0.01, *** *P* < 0.001.

Much evidence has demonstrated that aerobic glycolysis is frequently elevated in many cancer cells to support the malignant phenotypes of cancer cells [24]. DRP1 exerts its oncogenic functions by activation of glycolysis in PC cells [25]. Given this, we speculated that DRP1‐increased metabolic rewiring in tumor cells was required for MMP12 expression. First, we systematically analyzed the correlation between *DRP1* and glycolytic‐related molecules from the GEPIA database. The data showed that *DRP1* mRNA was positively correlated with the mRNA expressions of *ALDOA* (Spearman correlation *r* = 0.2; *P* < 0.001), *ENO1* (Spearman correlation *r* = 0.36; *P* < 0.001), *GPI* (Spearman correlation *r* = 0.27; *P* < 0.001), *PFKL* (Spearman correlation *r* = 0.31; *P* < 0.001) and *PGAM2* (Spearman correlation *r* = 0.27; *P* < 0.001) (Fig. 6A). These results indicate that DRP1 modulates glycolysis via regulation of multiple genes in patients with HNC. Secondly, we tested whether the glycolytic effect raised by DRP1 was dependent on MMP12. The glucose consumption and lactate production were measured in DRP1‐depleted HNC cells with MMP12 overexpression. The data showed that overexpressing MMP12 in DRP1‐depleted SAS and HSC‐3 cells reversed the *siDRP1* decrease in glucose consumption and lactate production in the culture medium (Fig. 6B,C). Finally, we examined the expression levels of glycolytic genes that correlated with DRP1 in HNC samples, as shown in Fig. 6A. Intriguingly, the transcriptional levels of *ALDOA*, *ENO1*, *GPI*, *PFKL* and *PGAM2* were generally upregulated in *siDRP1*‐HNC transfectants with MMP12 overexpression, compared with *siDRP1*‐HNC transfectants with vector alone (Fig. 6D). Taken together, DRP1 could potentiate glycolysis via MMP12 in HNC.

### 3.7 FOXM1 modulates the expression and function of MMP12 in HNC

**Fig. 7:**
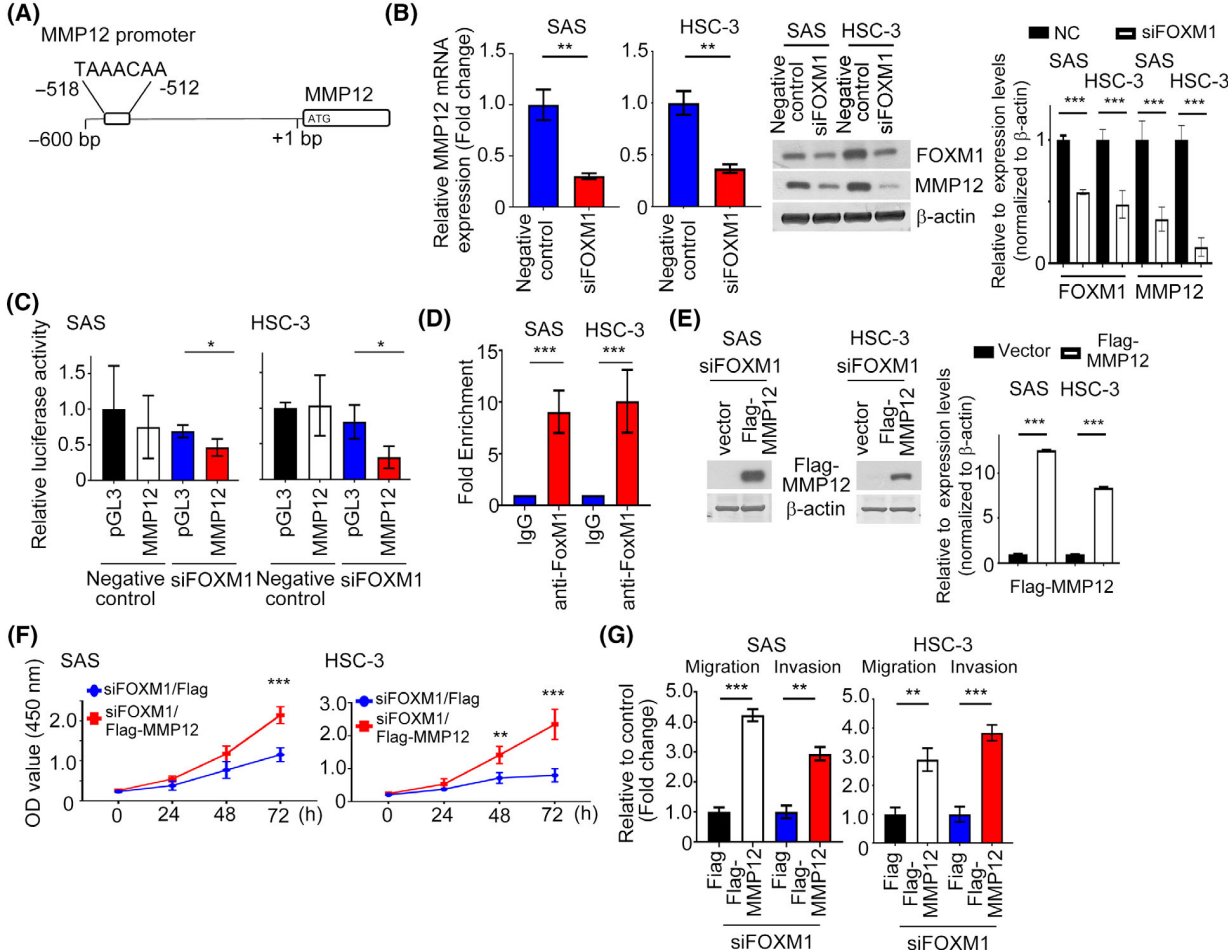
FOXM1 regulates the expression and transcription activity of MMP12 in HNC cells. (A) Potential FOXM1 binding sites in human *MMP12* promoter region. (B,C) QRT‐PCR, western blotting and luciferase assays indicating the expression and luciferase activity of MMP12 in FOXM1‐depleted HNC cells. Quantification of relative FOXM1 and MMP12 expressions are shown. (D) ChIP assays were performed to confirm the binding of FOXM1 to the *MMP12* promoter in SAS and HSC‐3 cells using an anti‐FOXM1 antibody. Isotype IgGs were used as a negative control. (E) The protein expression level of MMP12 was investigated in FOXM1‐depleted cells transfected with Flag‐MMP12 by western blotting. Quantification of relative Flag‐MMP12 expression is shown. (F,G) The growth and motility of *siFOXM1* with MMP12 overexpression in HNC transfectants was determined. All data are presented as mean  ±  SD of three independent experiments. Significance was calculated using *t*‐test. In (F), statistical analyses were performed using one‐way ANOVA followed by Tukey’s multiple comparison’s test. * *P* < 0.05, ** *P* < 0.01, *** *P* < 0.001.

Since MMP12 promoter activity was able to be upregulated by DRP1, we next tried to identify the possible transcriptional factors regulating MMP12 promoter activity. Our previous data indicated that FOXM1, a transcriptional factor, is overexpressed in HNC and its expression is correlated with poor survival of patients [26]. Additionally, high FOXM1 expression is involved in the migration and invasion of oral cancer cells, implying that FOXM1 plays an important role in regulating the aggressive behavior of cancer cells. Bioinformatics analysis indicated that a FOXM1 binding site was localized in a region between −518 and −512 bp on the *MMP12* promoter (Fig. 7A). Hence, we suggested FOXM1 could act on the *MMP12* promoter and regulate *MMP12* expression. FOXM1 inhibition by FOXM1‐mediated siRNA significantly decreased MMP12 mRNA and protein expression levels and *MMP12* luciferase activity in HNC cell lines by QPCR, western blotting and luciferase assays (Fig. 7B,C). Using ChIP assay, FOXM1 was seen to bond directly to the *MMP12* promoter in SAS and HSC‐3 cell lines (Fig. 7D). Functionally, MMP12 overexpression in FOXM1‐depleted SAS or HSC‐3 cells reversed siFOXM1‐attenuated cell growth, migration and invasion (Fig. 7E–G).

### 3.8 Knockdown of DRP1 alleviates miR‐575 inhibitor‐mediated glycolysis and mitochondria dysfunction

To determine whether miR‐575 could elicit its inhibitory effects by at least suppressing DRP1/FOXM1/MMP12 signaling, we transfected the miR‐575 inhibitor into *siDRP1*‐SAS cells. First, reduced FOXM1 and MMP12 protein levels were found in DRP1‐depleted SAS cells with miR‐575 inhibitor transfection, compared with negative control cells transfected with miR‐575 inhibitor (Fig. [Link], [Link], [Link], [Link], [Link], [Link], [Link]A). Functionally, blocking DRP1 signaling reversed miR‐575 inhibitor‐induced glucose consumption and lactate production in the culture medium (Fig. [Link], [Link], [Link], [Link], [Link]B). These data indicate that DRP1/FOXM1/MMP12 signaling plays an essential function during miR‐575 inhibition of metabolic features of HNC cancer cells. Dysfunction in mitochondrial dynamics results in alteration of reactive oxygen species (ROS) and ATP synthesis. Next, we investigate the effect of miR‐575/ DRP1 signaling on mitochondria function. Our data revealed that the decreased ROS production and increased ATP content were found in negative control‐SAS cells with miR‐575 inhibitor transfection, as compared with DRP1‐depleted cells with miR‐575 inhibitor transfection (Fig. [Link], [Link], [Link], [Link]C,D). The above data suggest that miR‐575‐mediated mitochondrial function is associated with DRP1 expression.

### 3.9 DRP1‐induced MMP12 expression is required for FOXM1

**Fig. 8:**
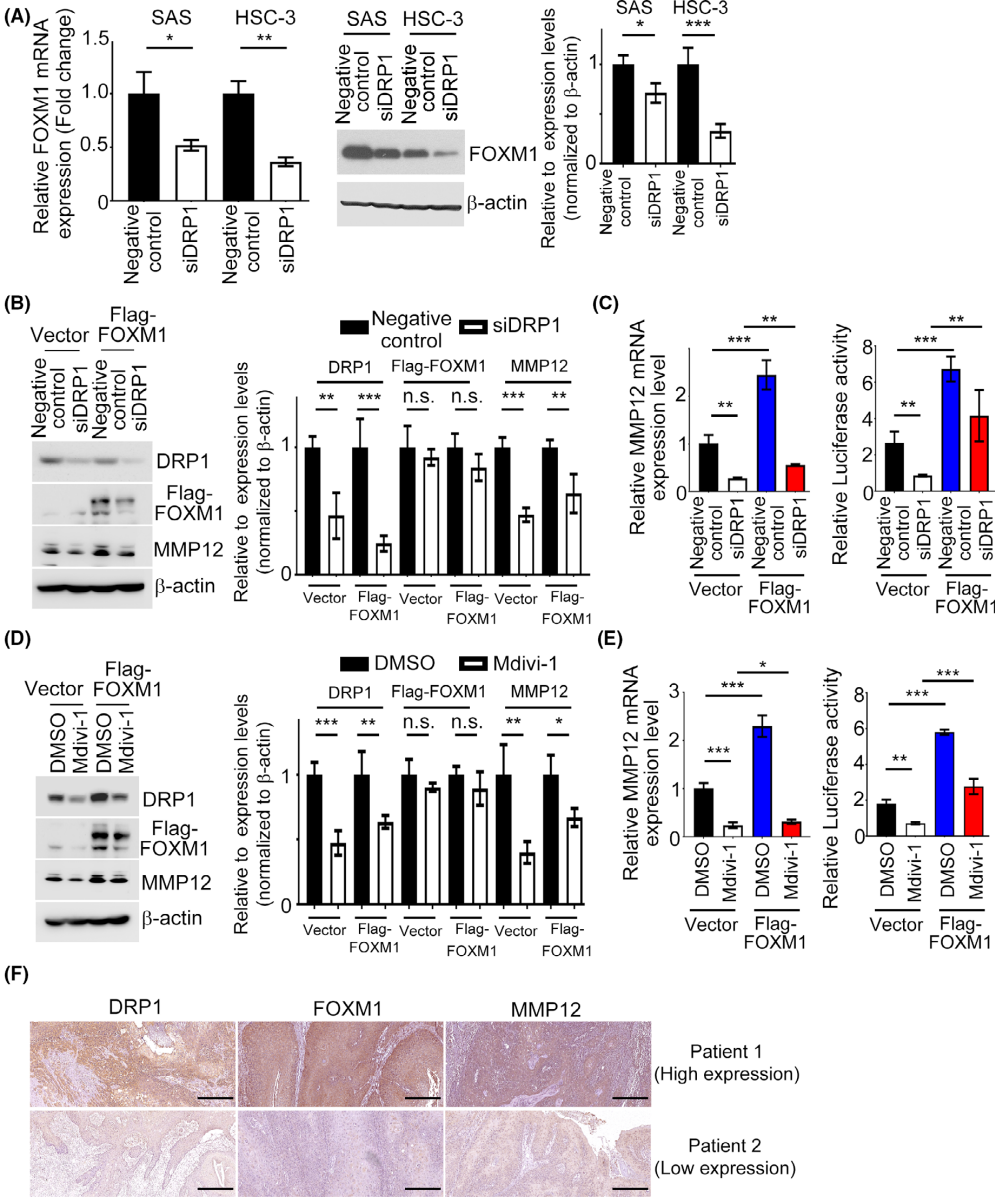
MMP12 expression is essential for DRP1/FOXM1 regulation in HNC cells. (A) The mRNA and protein expression levels of FOXM1 in *siDRP1* cells were examined. Quantification of relative FOXM1 expression is shown. (B,C) Western blotting, QPCR and luciferase activity analysis of MMP12 were determined in SAS cells transfected with FOXM1 or vector control in combination with *siDRP1* or negative control. Quantification of relative DRP1, Flag‐FOXM1 and MMP12 expressions is shown. (D,E) Western blotting, QPCR and luciferase activity of MMP12 were analyzed in SAS cells transfected with FOXM1 or vector control in combination with Mdivi‐1 treatment. Quantification of relative DRP1, Flag‐FOXM1, and MMP12 expressions is shown. (F) IHC staining patterns of the HNC tumor tissues for DRP1, FOXM1 and MMP12. Scale bar: 100 µm. All data presented as mean  ±  SD of three independent experiments. Significance calculated using *t*‐test. * *P* < 0.05, ** *P* < 0.01, *** *P* < 0.001.

We next validated the effect of DRP1 disruption on FOXM1 expression using QPCR and western blotting analysis; mRNA and protein levels of FOXM1 were obviously decreased in DRP1‐depleted HNC cells (Fig. 8A), indicating that FOXM1 expression could be regulated by DRP1 in HNC cells. In the GEPIA database, a positive correlation between *DRP1* and *FOXM1* in HNC tumor tissues was verified (Fig. [Link], [Link], [Link], [Link], [Link], [Link], [Link]). Furthermore, to investigate whether FOXM1 mediation of the expression of MMP12 is required for DRP1, vector expressing FOXM1 was introduced into SAS cells with DRP1 knockdown or negative control. Ectopic expression of FOXM1 rescued the decreased MMP12 protein, mRNA and luciferase activity in the SAS cells caused by DRP1 disruption (Fig. 8B,C). Consistently, similar results were observed in forced expression of FOXM1 in SAS cells treated with Mdivi‐1 (Fig. 8D,E). These data indicate that FOXM1‐dependent transcription is modulated by DRP1 in HNC cells. Subsequently, a positive association among DRP1, FOXM1 and MMP12 protein levels was confirmed in the tissue microarray using immunohistochemical staining (Fig. 8F). Among the 50 cases of HNC tissues, a positive correlation between the expression levels of DRP1 and FOXM1 proteins (correlation coefficient *r* = 0.722, *P* < 0.001), DRP1 and MMP12 proteins (correlation coefficient *r* = 0.634; *P* < 0.001), and FOXM1 and MMP12 proteins (correlation coefficient *r* = 0.74; *P* < 0.001) by Spearman’s correlation analyses were observed (Table 4). Collectively, these data demonstrate that FOXM1 binds to the MMP12 promoter and enhances its transcription in a DRP1‐dependent fashion.

**Table 4:** The correlation among DRP1, FOXM1and MMP12 protein expression in HNC tumor tissues.

	DRP1	FOXM1	MMP12
DRP1		Spearman’s correlation: *r* = 0.722	Spearman’s correlation: *r* = 0.634
		*P* < 0.001	*P* < 0.001
		*n* = 50	*n* = 50
FOXM1	Spearman’s correlation: *r* = 0.722		Spearman’s correlation: *r* = 0.74
	*P* < 0.001		*P* < 0.001
	*n* = 50		*n* = 50
MMP12	Spearman’s correlation: *r* = 0.634	Spearman’s correlation: *r* = 0.74	
	*P* < 0.001	*P* < 0.001	
	*n* = 50	*n* = 50	

## 4 Discussion

We provide evidence that the cellular level of DRP1 is significantly increased in HNC specimens and that its expression level is positively correlated with patient survival, indicating a function for DRP1 in HNC tumorigenesis. Inhibition of *DRP1* by *DRP1*‐mediated siRNA or Mdivi‐1 decreased HNC cell growth and metastasis *in vitro* and *in vivo*. The miR‐575 directly targeted *DRP1* and suppressed DRP1 mRNA and protein expression in HNC cell lines. A gene expression signature array disclosed that *DRP1* knockdown mainly impacted on *MMP12*, an extracellular matrix gene. MMP12 overexpression reversed the proliferative, migration, invasive and glycolytic effects of *DRP1* inhibition in HNC cells. FOXM1 was identified to bind directly to the *MMP12* promoter in order to induce its expression. The aggressive phenotypes of HNC cells elicited by FOXM1 were dependent on MMP12 expression. In a clinical study, *DRP1* expression was associated with *FOXM1* and *MMP12* expressions in HNC tumor tissues. We demonstrated that DRP1 modulates HNC development via a previously unexplored mechanism involving miR‐575/FOXM1/MMP12 signaling.

It is consistent with our clinical findings that DRP1 overexpression has been found in a variety of human cancers such as breast cancer [18], lung cancer [15, 27], ovarian cancer [28] and melanoma [29]. The overexpression of DRP1 and its nuclear localization caused lung adenocarcinoma cell drug resistance [27]. DRP1 was also highly expressed in human invasive breast carcinoma with lymph nodes metastases; conversely, silencing DRP1 resulted in significantly suppressed breast cancer metastasis. Interestingly, DRP1‐mediated mitochondrial fission played a critical role in cancer cell migration and invasion [18]. *In vitro* and *in vivo* studies reported reduced cancer cell growth, motility and/or enhanced spontaneous apoptosis induced by inhibiting DRP1 in several cancer types, including colon, breast and cervical cancers [15, 30, 31], indicating that targeting dysregulated DRP1 may provide a novel strategy for suppressing cancer growth and metastasis. Our findings are promising and provide important insights into developing novel prognostic marker and targeted therapy in patients with head and neck cancer.

Mdivi‐1, a cell‐permeable derivative of quinazolinone, was identified from chemical compound screening using yeast‐base assays [32]. Upon further analysis, Mdivi‐1 was a first selective inhibitor of mitochondrial fission that is reported to inhibit DRP1 in yeast and induce mitochondrial hyperfusion and elongation in mammalian cells [33]. Studies have shown that Mdivi‐1 alters mitochondria morphology, enhanced chemotherapy‐induced apoptosis in several cancer cells, induced genomic instability, and limited tumor size in cell lines and xenograft models [31, 34, 35, 36]. Glioblastomas and breast cancers treated with Mdivi‐1 were able to abolish their migratory and invasion abilities efficiently [37]. Additionally, treatment with Mdivi‐1 in breast, lung and skin cancers resulted in reduction of tumor‐sphere formation capability [38]. In the present results, we found that Mdivi‐1 attenuated proliferation, migration and invasion of head and neck cancer cells *in vitro* and *in vivo*. Furthermore, Mdivi‐1 inhibited the translational level of DRP1, thereby blocking the DRP1/FOXM1/MMP12 pathway. Taken together, these results lead us to conclude that the suppression of tumor growth and metastasis in head and neck cancer by Mdivi‐1 may partly be dependent on DRP1. However, it is worth noting that Mdivi‐1 has been shown to have off‐target effects involved in the inhibition of electron transport chain, indicating that antitumor effects of Mdivi‐1 are probably independent of DRP1 [39, 40]. Therefore, several proof‐of‐concept experiments are still required to understand the potential application in the clinical treatment of head and neck cancer.

MicroRNA, small non‐protein‐coding RNA, are plentiful in organisms and can bind to the 3’ UTR of the target mRNA, thus leading to inhibition of translational or mRNA degradation [41]. MicroRNA play a crucial roles in tumorigenesis, tumor progression and metastasis and serve as biomarkers for diagnosis and treatment response [42]. Recently, miR‐575, a member of miRNA, has attracted attention due to its biological role in many human cancers. Abnormal expression of miR‐575 may lead to cancer development. Previous studies indicated that miR‐575 plays an oncogenic role in promoting cell proliferation, migration and invasion in NSCLC, HCC and gallbladder cancer [22, 43, 44]. In contradiction to these results, miR‐575 overexpression significantly inhibited migration, proliferation and angiogenesis as well as induction of apoptosis of HUVEC by targeting the Rab5/MEK pathway, indicating that miR‐575 plays a tumor suppressor role in HUVEC [45]. In line with our finding, miR‐575 might act a tumor suppressor in HNC cells. This is the first report uncovering the underlying mechanism of miR‐575 in HNC cells which prevented cell proliferation, migration and invasion by suppressing its target gene DRP1, thus provide a new viewpoint for HNC treatment in the future.

FOXM1 is a transcriptional factor that plays a pivotal role in cancer development. FOXM1 is overexpressed in many human cancers and is associated with a poor prognosis, such as breast, oral cavity squamous cell carcinoma, head and neck, bladder, esophageal and colorectal cancers [24, 26, 46, 47, 48, 49]. FOXM1 is a crucial mediator in transduction of signals to downstream effectors in human cancer to influence cancer‐related processes, such as proliferation, migration, invasion, angiogenesis and EMT [51, 52, 53, 54]. For example, FOXM1 promotes cyclin A2, B1, D1 and VEGF transcription to modulate the cell‐cycle progression and angiogenic ability of cancer cells [25, 55]. Conversely, inhibition of FOXM1 reduced cell growth and contributed to chemosensitivity in cancer cells [56, 57, 58], indicating that targeting FOXM1 may be a promising strategy for cancers. In our current study, we demonstrate that the mRNA expression of FOXM1 was positively correlated with DRP1 expression in HNC specimens. In addition, FOXM1 expression was regulated by DRP1 in HNC cells, indicating that FOXM1 is one of the downstream targets of DRP1.

We next sought to examine whether DRP1 can interact with FOXM1, Co‐IP assays using antibodies against endogenous DRP1 and FOXM1 in SAS cells were performed. We did not detect an interaction between DRP1 and FOXM1 in the current experimental conditions (data not shown). Our results identified FOXM1 as a novel player in regulating DRP1 mechanism.

MMP12, also known as macrophage metalloelastase (MME) or macrophage elastase (ME), is an enzyme that take part in the decomposition of extracellular matrix in disease processes. MMP12 has an ability to degrade elastin to modulate tissue fibrosis [59]. Mice with MMP12 knockout showed markedly increased M2 macrophage accumulation [60], decreased sensitivity to cigarette smoke and attenuated elastin‐induced airway inflammation [61]. A recent study showed that extracellular MMP12 inhibition could be a therapeutic strategy for antiviral treatment [62]. MMP12 is not only expressed in macrophages, but is also found in tumor cells. A growing body of evidence shows that MMP12 dysregulation is involved in the development and progression of malignancies. MMP12 is upregulated in a variety of cancers and its expression is correlated with poor prognosis, such as gastric, liver, intestinal and lung cancers [63, 64, 65, 66, 67]. More importantly, enhanced expression of MMP12 is correlated with local recurrence and metastatic disease in NSCLC patients and with glioma and endometrial adenocarcinoma cell invasion [68, 69]. In line with our report, high MMP12 expression was found in tumor tissues of the HNC sample than in normal tissue in a publicly available cancer database, suggesting that MMP12 expression is involved in cancer progression of HNC. In contrast, some reports have indicated that MMP12 expression is associated with better prognosis in human cancers, such as colon, gallbladder and HCC cancers [67, 70]. Knocking out MMP12 promotes carcinogenesis in Apc^Min/+^ mice. These discrepant results indicate that the biological function of MMP12 in tumor progression differs between specific tumor cell types.

Prior work demonstrated that MMP12 activation facilitates the migration and invasion of chondrosarcoma through IGF‐1 and VEGF signaling pathways [70]. Lv et al. illustrated that MMP12 inhibition remarkably suppresses cell growth and invasion via downregulation of PCNA and VEGF in lung adenocarcinoma [71]. Our work for the first time shows that MMP12 expression was modulated by DRP1 involved in cell growth, migration and invasion in HNC cells. Extracellular MMP12 was also reduced in DRP1‐depleted HNC cells. We also identified a FOXM1 binding motif in the promoter sequence of MMP12. Furthermore, ChIP and biochemical assays demonstrated that FOXM1 bound to the MMP12 promoter regulates transcription of MMP12. Enforced MMP12 expression in FOXM1‐depleted HNC cells reversed *siFOXM1*‐decreased cell growth and motility. These results support our hypothesis that malignant phenotypes of HNC are elicited by DRP1 through FOXM1/MMP12 signaling.

## 5 Conclusion

To summarize, this study clearly demonstrates the pathological role of DRP1 in HNC, indicating that DRP1 serves as a promising prognostic factor in HNC. Meanwhile, DRP1 is validated to be a downstream target of miR‐575 and its expression is negatively correlated with the level of miR‐575 in HNC cells. The newly identified miR‐575/DRP1/FOXM1/MMP12 axis may be a potential therapeutic strategy for the intervention of HNC in clinic.

## Conflict of interest

The authors declare no conflicts of interest.

## Ethics approval and consent to participate

This study was approved by the Medical Ethics Committee and Animal Use and Management Committee of Chang Gung Memorial Hospital.

## Author contributions

TLH, CRC, YSL, FMF and CHC: experimental design and manuscript draft. CYC and GKH: clinical sample collection, interpretation and analysis. HTT, YFC and LJS: experimental manipulation and analysis of the results related to these assays. All authors read and approved the manuscript.

## Supporting information


**Fig. S1**. DRP1 contributed to tumor development in HNC.

**Fig. S2**. Inhibition of DRP1 decreased the migratory and metastatic abilities of SAS and HSC‐3 cells.

**Fig. S3**. Inhibition of endogenous DRP1 in miR‐575 mimics transfectant prevented the growth, migration and invasion in SAS and HSC‐3 cells elicited by miR‐575 mimics

**Fig. S4**. MMP12 expression was determined in DRP1‐depleted HNC cells.

**Fig. S5**. MMP12 expression level and its expression relative to DRP1 in HNC samples are shown.

**Fig. S6**. miR‐575 inhibitor activated the DRP1/FOXM1/MMP12 pathway and mediated mitochondrial function.

**Fig. S7**. DRP1 expression level was correlated with FOXM1 expression in HNC samples.

## Data Availability

All data generated or analyzed during this study are included in this published article.
