# Supplementary figures and images for: DRP1 contributes to head and neck cancer progression and induces glycolysis through modulated FOXM1/MMP12 axis
Source: Mol Oncol. 2022 Apr 15;16(13):2585–606. doi: 10.1002/1878-0261.13212 (PMC9251862; doi:10.1002/1878-0261.13212)

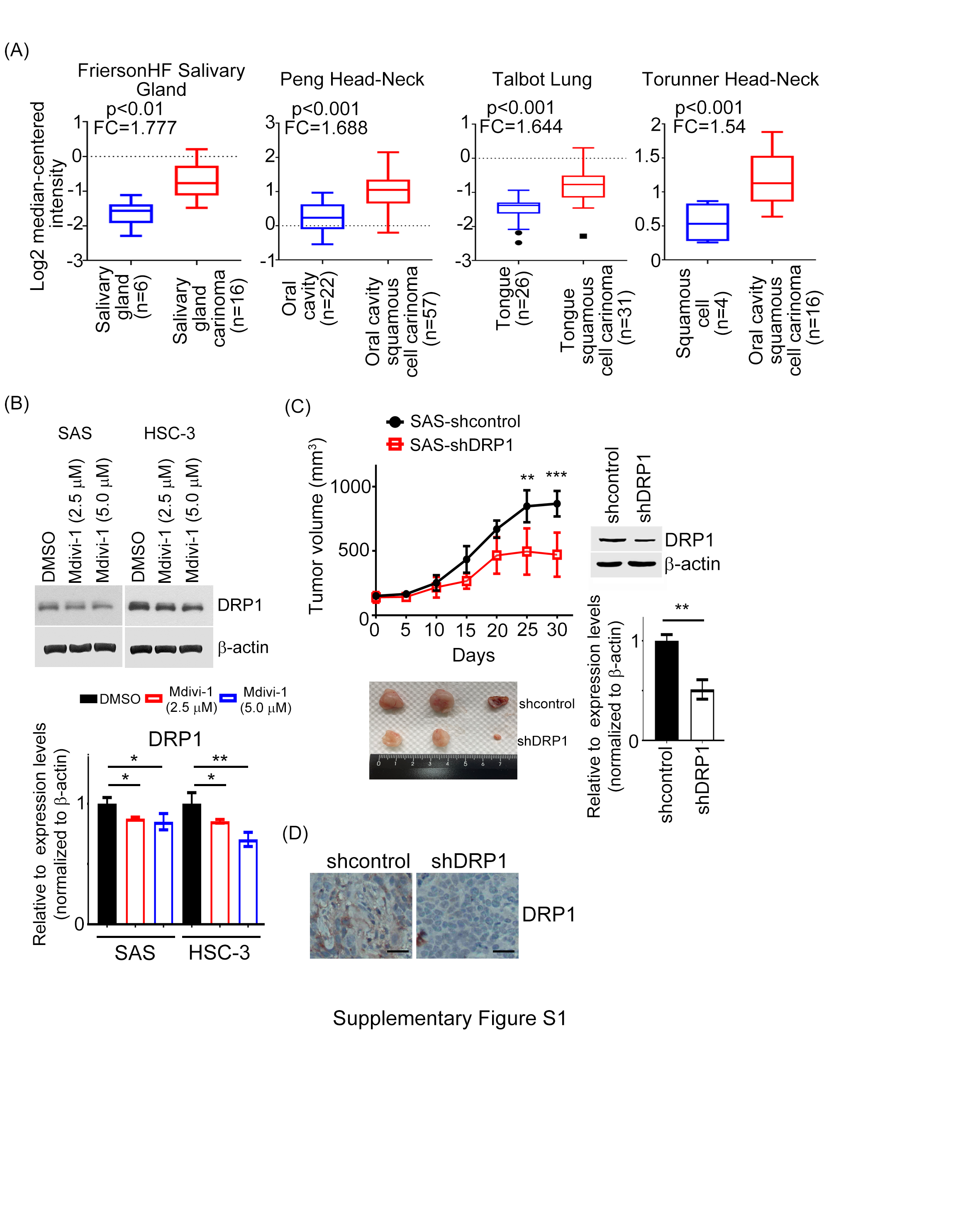

Supplement: Supplementary file 1 — Fig. S1. DRP1 contributed to tumor development in HNC. [file MOL2-16-2585-s006.tif]

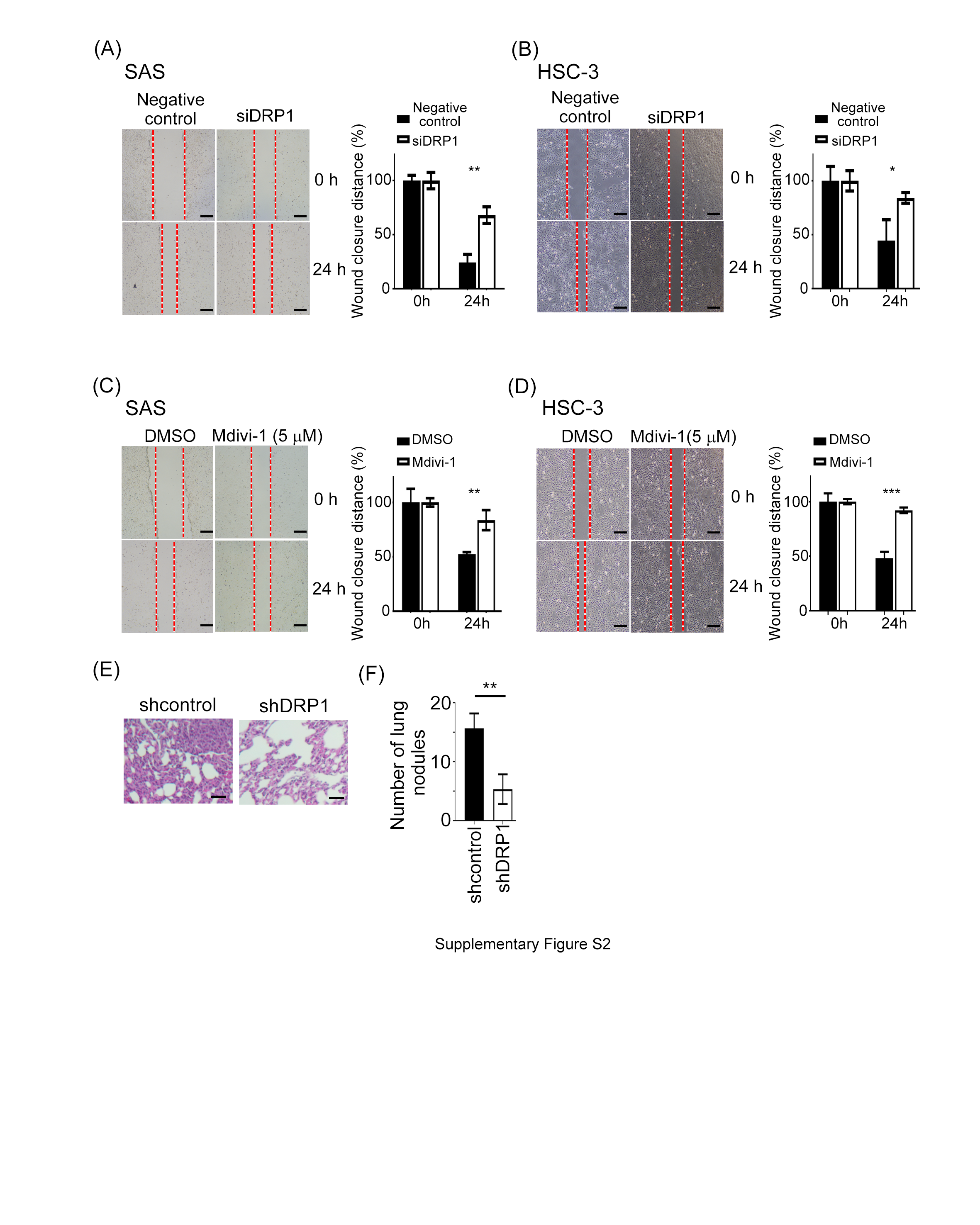

Supplement: Supplementary file 2 — Fig. S2. Inhibition of DRP1 decreased the migratory and metastatic abilities of SAS and HSC‐3 cells. [file MOL2-16-2585-s004.tif]

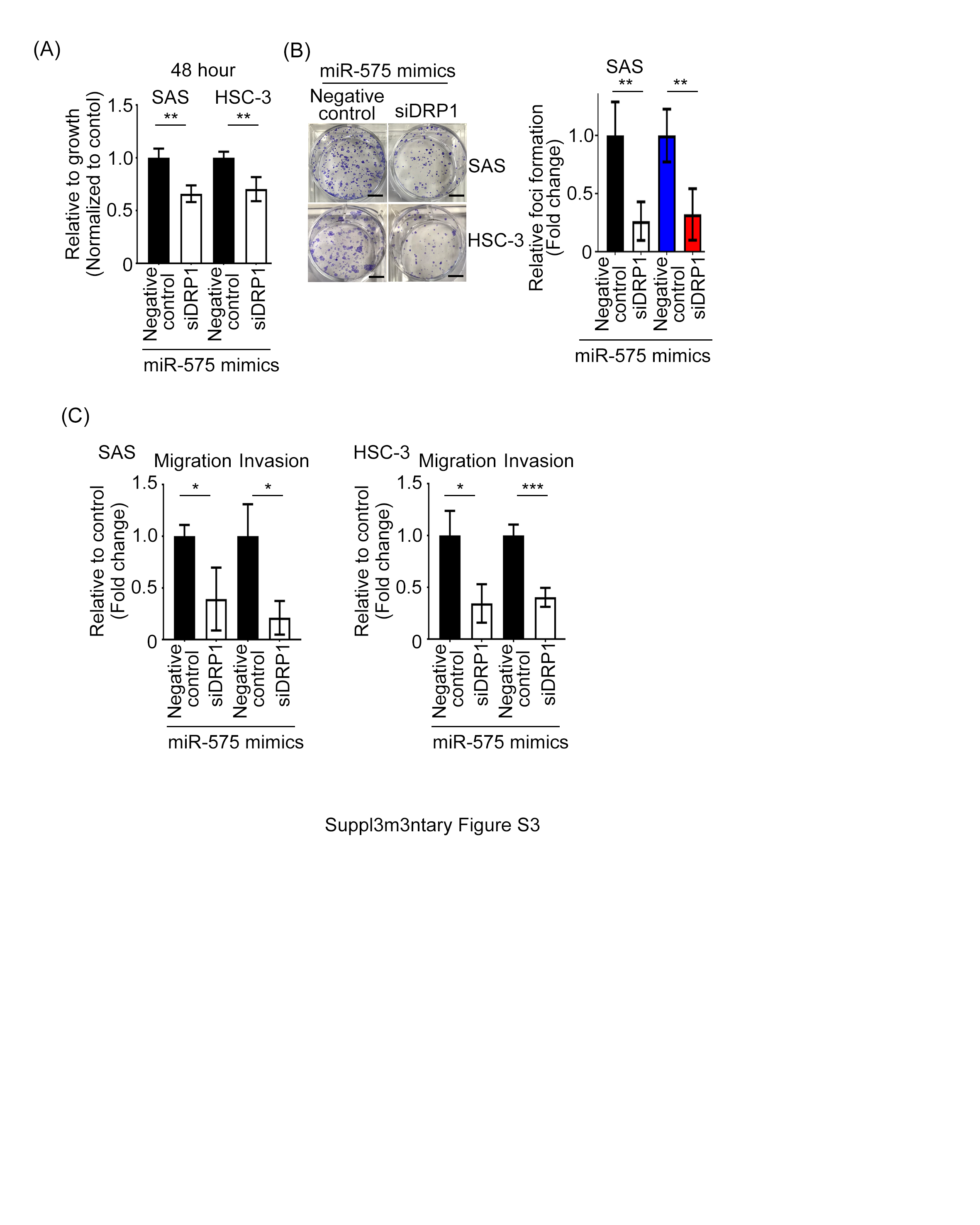

Supplement: Supplementary file 3 — Fig. S3. Inhibition of endogenous DRP1 in miR‐575 mimics transfectant prevented the growth, migration and invasion in SAS and HSC‐3 cells elicited by miR‐575 mimics [file MOL2-16-2585-s005.tif]

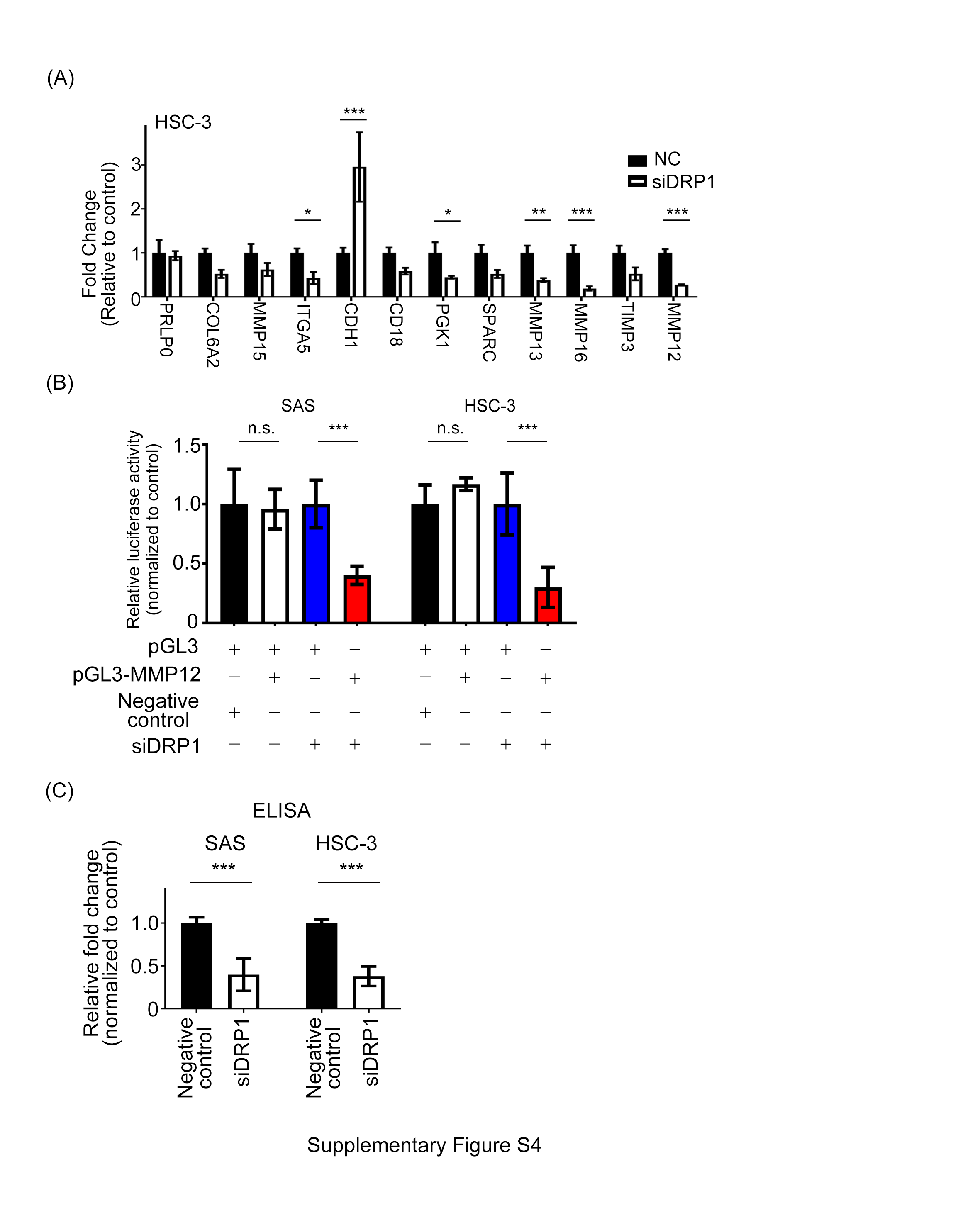

Supplement: Supplementary file 4 — Fig. S4. MMP12 expression was determined in DRP1‐depleted HNC cells. [file MOL2-16-2585-s002.tif]

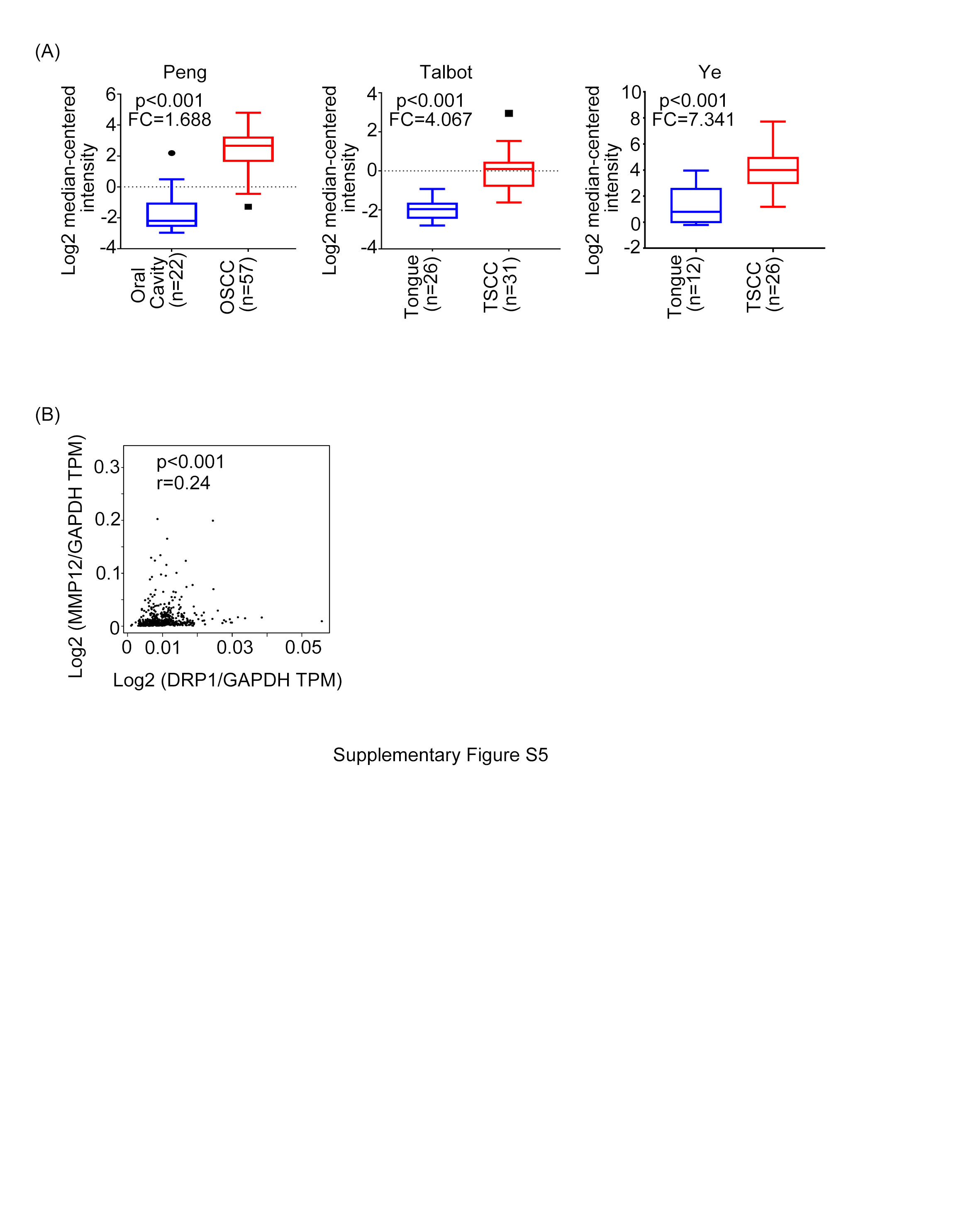

Supplement: Supplementary file 5 — Fig. S5. MMP12 expression level and its expression relative to DRP1 in HNC samples are shown. [file MOL2-16-2585-s007.tif]

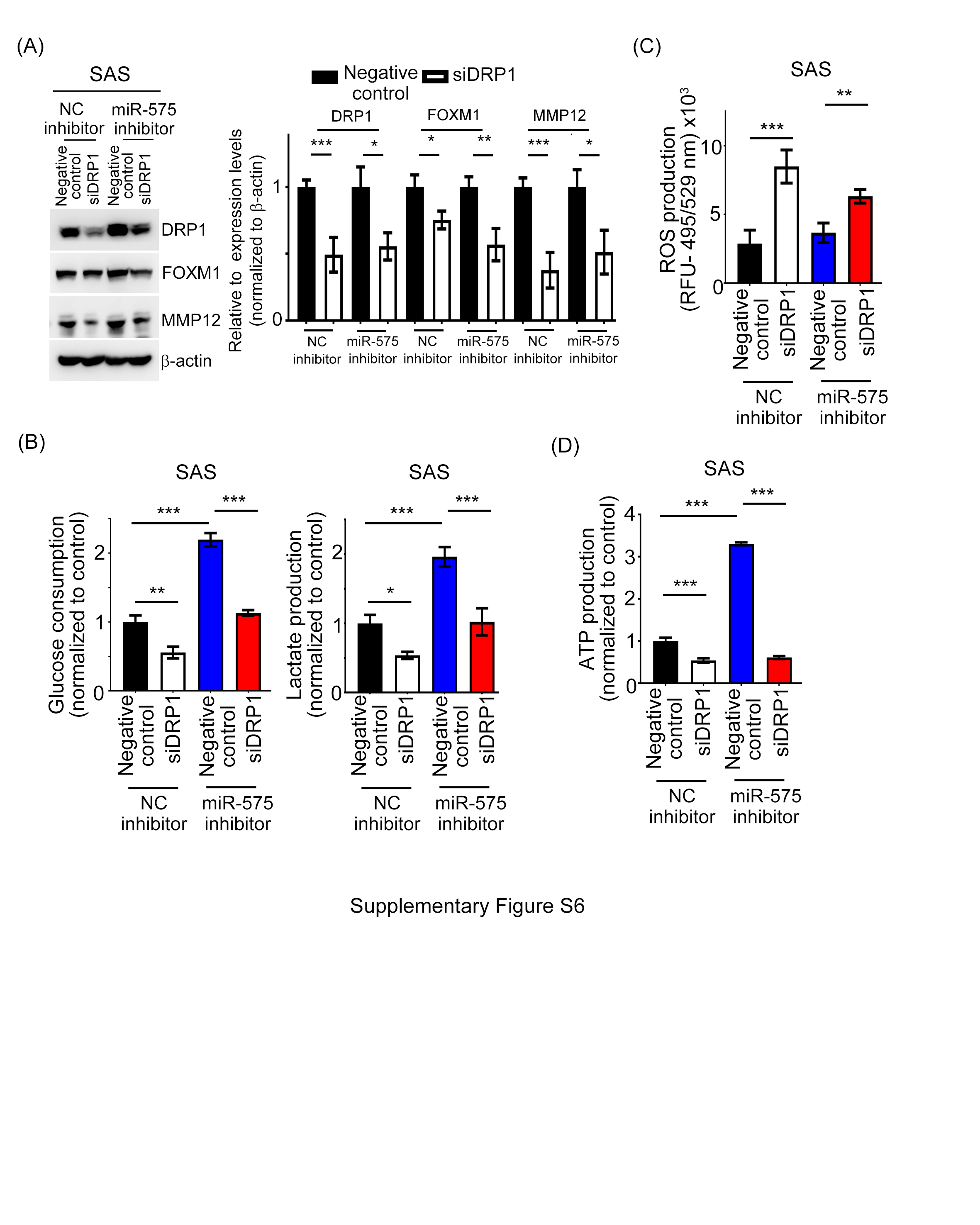

Supplement: Supplementary file 6 — Fig. S6. miR‐575 inhibitor activated the DRP1/FOXM1/MMP12 pathway and mediated mitochondrial function. [file MOL2-16-2585-s003.tif]

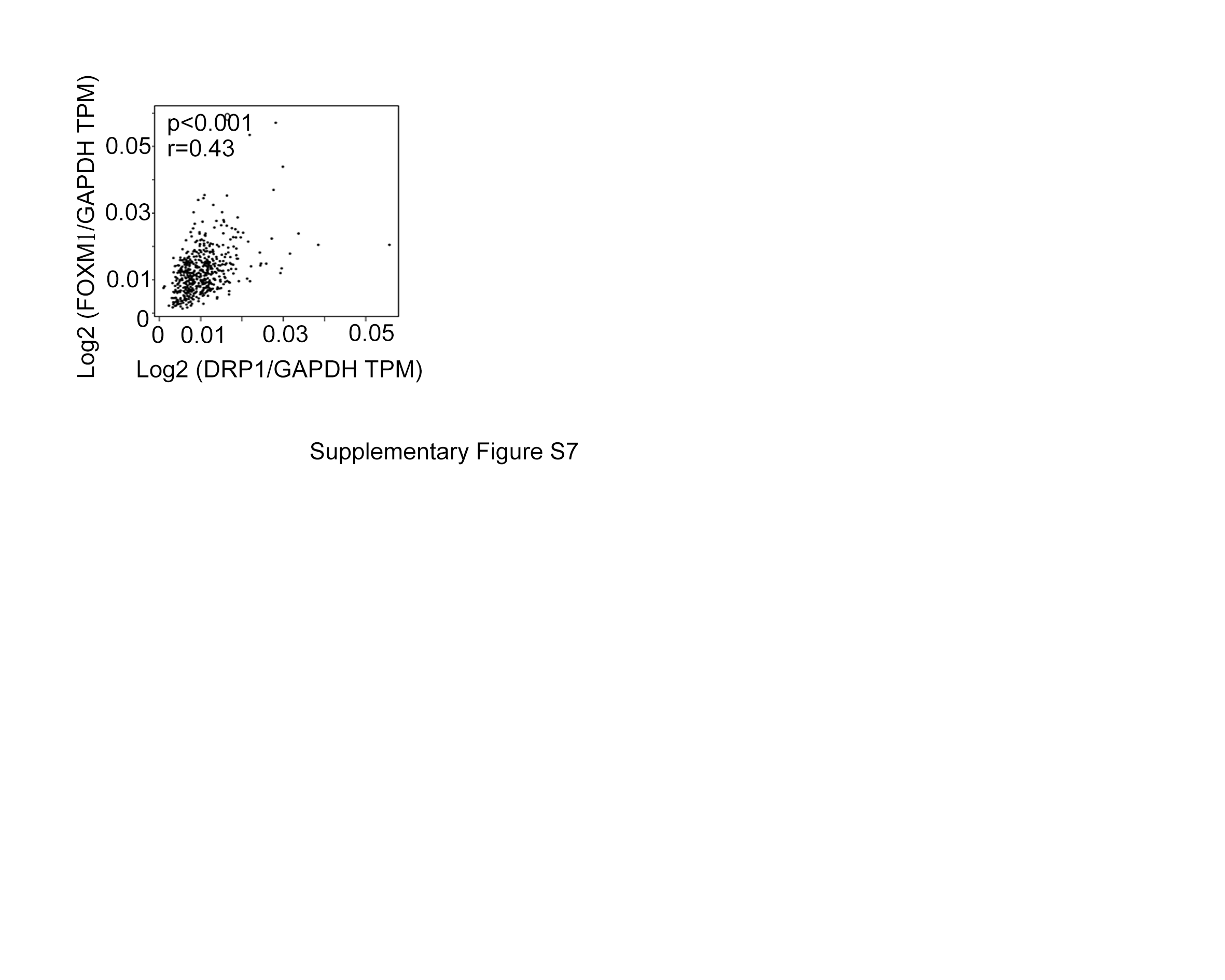

Supplement: Supplementary file 7 — Fig. S7. DRP1 expression level was correlated with FOXM1 expression in HNC samples. [file MOL2-16-2585-s001.tif]
